# Adaptive neuromodulation dialogues: navigating current challenges and emerging innovations in neuromodulation system development

**DOI:** 10.1088/1741-2552/ae2359

**Published:** 2025-12-19

**Authors:** Frederik Lampert, Matthew R Baker, Michael A Jensen, Amir H Ayyoubi, Christian Bentler, Jessica L Bowersock, Rosana Esteller, Jeffrey A Herron, Graham W Johnson, Daryl R Kipke, Christopher K Kovach, Vaclav Kremen, Filip Mivalt, Joseph S Neimat, Theoden I Netoff, Enrico Opri, Alexander Rockhill, Joshua M Rosenow, Kristin K Sellers, Nathan P Staff, Chandra Prakash Swamy, Ashwin Viswanathan, Gerwin Schalk, Timothy Denison, Dora Hermes, Nuri F Ince, Peter Brunner, Gregory A Worrell, Kai J Miller

**Affiliations:** 1 Department of Neurosurgery, Mayo Clinic, Rochester, MN, United States of America; 2 Medical Scientist Training Program, Mayo Clinic, Rochester, MN, United States of America; 3 Department of Bioinformatics and Computational Biology, University of Minnesota, Minneapolis, MN, United States of America; 4 Department of Biomedical Engineering, Mayo Clinic, Rochester, MN, United States of America; 5 CorTec GmbH, Freiburg, Germany; 6 Department of Neurological Surgery, University of Louisville, Louisville, KY, United States of America; 7 Boston Scientific: Neuromodulation, Research and Advanced Concepts Team, Valencia, CA, United States of America; 8 Department of Neurological Surgery, University of Washington, Seattle, WA, United States of America; 9 Department of Biomedical Engineering, Vanderbilt University, Nashville, TN, United States of America; 10 NeuroNexus Technologies, Ann Arbor, MI, United States of America; 11 University of Nebraska Medical Center, Omaha, NE, United States of America; 12 Department of Neurology, Mayo Clinic, Rochester, MN, United States of America; 13 Department of Biomedical Engineering, University of Minnesota, Minneapolis, MN, United States of America; 14 Department of Biomedical Engineering, University of Michigan College of Engineering, Ann Arbor, MI, United States of America; 15 Departments of Neurological Surgery, Oregon Health & Science University, Portland, OR, United States of America; 16 Department of Neurosurgery, Northwestern University Feinberg School of Medicine, Chicago, IL, United States of America; 17 Department of Neurosurgery, University of California, San Francisco, CA, United States of America; 18 Department of Neurosurgery, Baylor College of Medicine, Houston, TX, United States of America; 19 Department of Electronic & Electrical Engineering, University of Bath, Bath, United Kingdom; 20 Department of Engineering Sciences, Oxford University, Oxford, United Kingdom; 21 Department of Neurosurgery, Washington University School of Medicine, St Louis, MO, United States of America

**Keywords:** adaptive neuromodulation, closed-loop stimulation, implantable brain-computer interface (iBCI), implantable neural stimulators (INS), deep brain stimulation (DBS)

## Abstract

Adaptive neuromodulation systems and implantable brain-computer interfaces have made notable strides in recent years, translating experimental prototypes into clinical applications and garnering substantial attention from the public. This surge in interest is accompanied by increased scrutiny related to the safety, efficacy, and ethical implications of these systems, all of which must be directly addressed as we introduce new neurotechnologies. In response, we have synthesized the insights resulting from discussions between groups of experts in the field and summarized them into five key domains essential to therapeutic device development: (1) analyzing current landscape of neuromodulation devices and translational platforms (2) identifying clinical need, (3) understanding neural mechanisms, (4) designing viable technologies, and (5) addressing ethical concerns. The role of translational research platforms that allow rapid, iterative testing of hypotheses in both preclinical and clinical settings is emphasized. These platforms must balance experimental flexibility with patient safety and clear clinical benefit. Furthermore, requirements for interoperability, modularity, and wireless communication protocols are explored to support long-term usability and scalability. The current regulatory processes and funding models are examined alongside the ethical responsibilities of researchers and device manufacturers. Special attention is given to the role of patients as active contributors to research and to the long-term obligations we have to them as the primary burden-bearers of the implanted neurotechnologies. This article represents a synthesis of scientific, engineering, and clinical viewpoints to inform key stakeholders in the neuromodulation and brain-computer interface spaces.

**Acronyms**
aDBS
: Adaptive deep brain stimulation
ALS
: Amyotrophic lateral sclerosis
ANT
: Anterior nucleus of the thalamus
API
: Application programming interface
BCI
: Brain–computer interface
iBCIs
: Implantable brain–computer interfaces
BIC
: Brain Interchange
BSEPs
: Brain stimulation-evoked potentials
BT
: Bluetooth
CCEPs
: Cortico-cortical evoked potentials
CED
: Coverage with evidence development
CMS
: Centers for Medicare & Medicaid Services
DBS
: Deep brain stimulation
DLEPs
: DBS local evoked potentials
ECoG
: Electrocorticography
EEG
: Electroencephalography
EMU
: Epilepsy monitoring unit
ERNA
: Evoked resonant neural activity
FDA
: U.S. Food and Drug Administration
GPi
: Globus pallidus internus
HFOs
: High-frequency oscillations
HIPAA
: Health Insurance Portability and Accountability Act
IDE
: Investigational device exemption
iEEG
: Intracranial electroencephalography
IMDs
: Implantable medical devices
INS
: Implantable neural stimulator
INSR
: Implantable neural stimulating and recording
LFPs
: Local-field potentials
MICS
: Medical Implant Communication Systems
MRI
: Magnetic resonance imaging
NFC
: Near-field communication
NCAN
: National Center for Adaptive Neurotechnologies
NIH
: National Institutes of Health
NQ
: Natus Quantum®
NWB
: Neurodata Without Borders
OCD
: Obsessive-compulsive disorder
ORs
: Operating rooms
PD
: Parkinson’s disease
PMA
: Premarket approval
RF
: Radio frequency
RNS
: Responsive neurostimulation
SCS
: Spinal cord stimulation
SOZ
: Seizure onset zone
SEEG
: Stereoelectroencephalography
STN
: Subthalamic nucleus
TMS
: Transcranial magnetic stimulation
WBANs
: Wireless body area networks

## 1. Introduction

The standard clinical management of neurological and psychiatric disorders predominantly relies on pharmacological interventions or, in select cases, irreversible surgical procedures, such as lesioning. While these approaches have shown efficacy in many conditions, they often lack specificity, carry the risk of systemic side effects, and may not adequately address the complex and dynamic nature of brain network dysfunctions [1, 2].

*Neuromodulation* has emerged as a promising therapeutic paradigm that offers a targeted, reversible intervention by altering neural activity through electrical, chemical, or magnetic means [1, 2]. Neuromodulation includes both invasive and noninvasive techniques that can target the central or peripheral nervous system [1, 3]. To narrow the scope, this article focuses on **invasive electrical neuromodulation targeting the central nervous system**, specifically technologies interfacing brain, such as DBS, RNS, and iBCIs. Although the broader term BCI includes non-invasive technologies (e.g. EEG-based systems), we use the term iBCIs to refer specifically to invasive, implantable systems with high-bandwidth data transmission and direct cortical interface [4].

Neuromodulation and iBCIs systems are increasingly used in clinical practice [2, 5], have become a major focus of academic and clinical research (see supplement), and have attracted growing financial and industry interest in developing devices that support bidirectional communication for adaptive personalized therapies [6–8].

This article summarizes the current neuromodulation landscape, including recent advances in clinical and research applications, an overview of available implantable neuromodulation and iBCIs devices, and key considerations for developing new therapeutic technologies.

One of the major challenges investigators and engineers face is the rigorous validation of their hypotheses and designs. Current research of novel therapies most often takes the form of *feed-forward* studies, where researchers propose a hypothesis and design a clinical trial to test whether a specific intervention improves patient outcomes. These hypotheses are often evaluated using a single clinical metric. In neuromodulation, they typically concern whether a particular hardware–software system positively impacts a certain aspect of the disease. This strategy has evolved from how the clinical efficacy of pharmaceuticals is tested. However, the complexity of neuromodulation often exceeds that of traditional pharmacological interventions, as only a specific combination of anatomical target and stimulation strategy (frequency, current, and timing) may yield the desired clinical benefits. Current regulatory procedures have created a situation in which researchers are forced to fully commit to a singular hypothesis, often without fully understanding or validating the mechanistic underpinnings of the proposed therapy. As a result, even technically sound and well-developed neuromodulation studies may fail to create the necessary clinical outcomes that justify continued clinical translation [9]. In addition, technological excellence alone does not guarantee clinical or commercial success. If a neuromodulation device fails to meet broader requirements, such as cost, usability, regulatory approval, or integration into an ecosystem, it is unlikely to be adopted, regardless of its scientific and clinical appeal [9, 10]. Overcoming these barriers requires creating an environment in which ideas can be rapidly and iteratively tested and refined before being subjected to lengthy and expensive clinical trials. This would provide better insight into the steps and mechanisms of the therapy, creating a higher chance for the study to succeed.

One promising path forward is the development of translational platforms—flexible systems designed to support rapid, iterative testing of hypotheses with minimal disruption to ongoing clinical care. Such platforms would bridge standard therapy and exploratory research, enabling a continuous feedback loop that benefits both scientific discovery and patient outcomes. This article highlights specific systems and platforms as representative examples to illustrate broader translational principles. Technologies were selected based on relevance, technical features, and visibility in the field. Where relevant, comparisons to similar tools are included to provide context within the wider technological landscape.

### 1.1. Motivation & objective

Recent publications have provided valuable overviews of the current state of iBCIs clinical trials [11, 12], including their use in neuropsychiatric disorders [13], ethical considerations [14], and broader reflections on the translation of neurotechnologies into practice [10, 15]. While these works provide important context, our goal is to build on them by offering more practical consensus-driven recommendations and considerations for designing neuromodulation and iBCIs technologies to accelerate clinical translation. It emphasizes modularity, interoperability, standardization, and the need for multidisciplinary collaboration to support real-time, hypothesis-driven development of novel therapies. To move from concept to implementation, the development of such systems requires broad consensus between disciplines. To support this goal, we convened a **symposium of experts** from the scientific, clinical, engineering, and regulatory communities to exchange ideas, discuss and define the key requirements these platforms must meet. This article is a direct outcome of those discussions and aims to provide an overview of the current landscape of neuromodulation and iBCIs technologies. In doing so, we highlight key considerations for advancing translational technologies, grounded in analyzing the current state of the field (section 2), shared clinical needs (section 3), neuroscientific understanding (section 4), technological considerations (section 5), and ethical (section 6.1) and regulatory frameworks (section 2.1 in supplement).

## 2. Current landscape of neuromodulation devices and translational platforms

Current neuromodulation therapies predominantly rely on *open-loop* high-frequency stimulation, with clinicians adjusting settings based on patient feedback [1, 2, 16–18]. The majority of clinical applications focus on the treatment of movement disorders, such as PD & essential tremor, chronic pain, and epilepsy [13, 17–24].

In recent years, the field has shifted its focus toward *closed-loop* or responsive stimulation systems, which deliver stimulation in response to real-time signals. This approach improves specificity, reduces side effects, and improves energy efficiency [16, 17]. *Closed-loop* adaptive systems are already used in the treatment of epilepsy [23, 25], and movement disorders [16, 17, 26]. Ongoing clinical trials explore their use in stroke rehabilitation [27, 28], chronic pain [24, 29, 30] and in neuropsychiatric conditions such as depression and OCD [13, 31, 32]. Although these developments mark important progress, treatment outcomes still vary between patients, and INS are often used only after other therapies have failed [15, 33–37]. This contributes to their limited adoption in mainstream clinical practice and underutilizes the full therapeutic potential of these technologies [10].

Parallel to developments in neuromodulation, iBCIs studies have shown promising results in restoring communication [38–40], movement [41, 42] and control over a device [43, 44]. Recent reviews by Patrick-Krueger *et al* [11] and Zhang *et al* [12] provide comprehensive overviews of current iBCI research and clinical studies. Despite recent advancements, iBCIs systems continue to face key challenges that hinder their clinical translation. These include limited portability, lack of generalizability across users, and inconsistent long-term performance, particularly as neural signals evolve over time due to factors such as gliosis, signal degradation, or user adaptation [11, 12]. These limitations complicate the development of systems that are robust enough for scalable, long-term clinical use. To address these barriers, there is a growing emphasis in the iBCIs field on developing adaptive algorithms that support self-calibration and dynamic learning, allowing systems to automatically adjust to intra and inter-subject variability [11, 12]. However, **no iBCIs system has yet received regulatory approval for therapeutic use** [45], highlighting the ongoing gap between research innovation and clinical implementation.

Beyond technical challenges, the development of both neuromodulation and iBCIs systems is shaped by logistical constraints, high development costs, and the need to balance technical readiness, clinical necessity, and economic viability [9, 10]. Active feedback from the neurotechnology community is essential in this process, underscoring the importance of effective communication channels. Progress in this space may be accelerated by standardizing translational workflows and platform architectures, which could help bridge the gap between early adopters and widespread clinical integration, ultimately bringing these technologies into mainstream therapeutic practice [2, 10].

### 2.1. Overview of neuromodulation and iBCI platforms

The neuromodulation and iBCIs field is rapidly evolving, with a growing number of devices in development. This section, together with table 1, provides an overview of current platforms from both established and emerging companies. The neuromodulation industry is currently led by three dominant medical device companies: Medtronic, Boston Scientific, and Abbott. These manufacturers provide the majority of DBS systems used clinically. Their platforms offer robust and programmable open-loop stimulation with varying degrees of programmability, power systems (rechargeable vs. non-rechargeable), and lead compatibility achieved through adapters [46]. For instance, Medtronic’s Percept^TM^ RC and Percept^TM^ PC systems provide 16-contact leads, continuous programmable stimulation, and LFPs sensing, with either rechargeable or non-rechargeable batteries depending on the model. In comparison, Abbott’s Liberta RC and Infinity^TM^ systems support similar electrode configurations but lack LFP sensing capabilities. Boston Scientific’s Vercise^TM^ family (Gevia, Genus P8/P16/R16/P32/R32) provides up to 32 contacts with programmable stimulation, some rechargeable models, and broad compatibility with leads via adapter systems [46]. While these companies dominate in Western markets, PINS Medical has emerged as a major neuromodulation player in Asia, particularly in China. PINS offers several CE-marked DBS systems (e.g. G102R, G102, G101A) designed for movement disorder therapies, featuring both rechargeable and non-rechargeable versions. Although major players continue to dominate clinical settings, emerging companies are trying to enter the market by adding adaptive stimulation and sensing capabilities. Startups and academic-industrial partnerships are driving innovation in this space. Their platforms are engineered with flexibility in mind, often using modular systems to support future development and wider applicability. Examples of such platforms are CorTec’s BIC, Cadence Neuroscience’s Alera, Newronika’s AlphaDBS, Picostim from Amber Therapeutics and Iris Biomedical’s Athena.

**Table 1.** List of the current commercial and research iBCIs & INS platforms.

Manufacturer	Device	Intended use	Regulatory approval	Number of contacts	Powering	Stim capability	LFP sensing	Lead Intercompatibility	State of the development
Abbott Laboratories	Liberta RC [89]	DBS for MD	FDA [90]	16 (2 × 8)	Rechargeable battery	Continuous programmable	No	Yes	Clinic
Abbott Laboratories	Infinity™ [91]	DBS for MD	FDA [90],CE [92]	16 (2 × 8)	Non-rechargeable battery (*⩾*5 years)	Continuous programmable	No	Yes (Medtronic)	Clinic
Amber Therapeutics	‘PicostimTM-DyNeuMo [93]	DBS for MD	IDE [93,94 ]	8 (2 × 4)	Rechargeable battery [93]	Adaptive	No	Yes (for DBS leads)	Pre-clinic
Blackrock Neurotech	Utah array	BCI	FDA 510(k) [50]	128 or 256	External	No	Yes	No	Pre-clinic
Boston scientific	Vercise™ PC	DBS for MD	FDA [95], CE [96])	16	Non-rechargeable battery	Continuous programmable*	No	Yes (by adapters [46])	Clinic
Boston scientific	Vercise™ Genus P8/P16/P32 [97]	DBS for MD	FDA [95],CE [96]	8/16/32	Non-rechargeable battery (3–5 years)	Continuous programmable*	No	Yes (by adapters [46])	Clinic
Boston scientific	Vercise™ Genus R16/R32	DBS for MD	FDA [95], CE [96]	16/32 [97]	Rechargeable	Continuous programmable*	No	Yes (by adapters[46])	Clinic
Boston scientific	Vercise Gevia™	DBS for MD	FDA [95], CE [98])	16	Rechargeable	Continuous programmable*	No	Yes (by adapters [46])	Clinic
Cadence neuroscience	Alera™	RNS for Epilepsy	No [99]	16 (4 × 4) [86]	Rechargeable [86]	Continuous programmable*	Yes (2 channels) [86]	Unknown (but modular)	Pre-clinic
Cleveland FES	NNP System	Multiple indications	FDA’s breakthrough	9635 [101]	Modular rechargeable battery [102]	Indirect	Limited (power in 300–1000 Hz) [102]	Partially (Utah array) [102]	Experimental device
			Medical device [100]						
CorTec	Brain Interchange	BCI, Stroke recovery	IDE [103]	32	External inductive	Adaptive	Yes	Yes	Pre-clinic
GenLight	Epilcure	Epilepsy [104]	No	8 (2 × 4) [105]	External inductive	Adaptive [106]	Partial	No (but modular)	Pre-clinic
Inbrain Neuroelectronics	BCI-Tx	BCI, PD	IDE [107]	Unknown	Unknown	Adaptive	Yes	Unknown	Pre-clinic
Iris Biomedical	Athena [87]	BCI	No	32 (4 × 8)	External Inductive	Adaptive	Yes	Yes	Pre-clinic
Medtronic	Activa™ RC	DBS for MD	FDA 510(k) [108](premarket)	16 (2 × 8)	Rechargeable battery	Continuous programmable	No	Yes	Clinic
Medtronic	Percept™ PC	DBS for MD, OCD, Epilepsy20	FDA 510(k) [109], CE [110]	16 (2 × 8)	Non-rechargeable battery (*⩾* years)	adaptive [55]	Yes	Yes	Clinic
Medtronic	Percept™ RC	DBS for MD, OCD, Epilepsy20	FDA 510(k) [109], CE [110]	16 (2 × 8)	Rechargeable battery	adaptive [55]	Yes	Yes	Clinic
Medtronic	Summit RC + S	Depression, epilepsy	IDE [30]	16 (2 × 8)	Rechargeable battery	Adaptive	Yes (4 channels)	Yes	Discontinued
Motif	Dot	Mental disorders	In process [111]	N/A	External Inductive	Adaptive TMS [47]	N/A	N/A	Pre-clinic
Neuralink	Link [51]	BCI	IDE [112]	1024	External Inductive	Adaptive	Yes	No	Pre-clinic
Neuropace	RNS [113]	Epilepsy	FDA 510(k) [52]	8 (2 × 4)	Non-rechargeable battery (*≈*11 years)	Adaptive	Yes (4 channels) [114]	No (but modular)	Pre-clinic
Newronika	AlphaDBS® [115]	DBS for MD	IDE [116]	16 (2x8)	Rechargeable battery	Adaptive	Yes (2 channels) [115]	Yes [117]	Pre-clinic
Onward Medical®	WIMAGINE	ECoG recordings	Clinical trial [118]	64 [42, 119, 120]	External Inductive	No	Yes	No	Pre-clinic
Paradromics	CONNEXUS® [121]	BCI	FDA’s Breakthrough	Up to 1684 (4x421)	External Inductive [121]	No	Yes	No (but modular)	Pre-clinic
			Medical Device [122]						
PINS Medical	G101A [123]	DBS for MD	CE [124]	4	Non-rechargeable battery	Continuous Programmable	No	No (but modular)	Pre-clinic
PINS Medical	G102 [125]	DBS for MD	CE [124]	8 (2 × 4)	Non-rechargeable battery	Continuous programmable	No	No (but modular)	Pre-clinic
PINS Medical	G102R [126]	DBS for MD	CE [124]	8 (2 × 4)	Rechargeable battery	Continuous programmable	No	No (but modular)	Pre-clinic
Precisis GmbH	EASEE® [127]	Epilepsy	Breakthrough Device [128]	5 [130]	Internal	Continuous (Adaptive in future) [130]	In next generation [130]	No (but modular)	Pre-clinic
			CE [129]						
Salvia Bioelectronics	MySalvia [49]	Chronic migraine	FDA’s Breakthrough	8	External inductive	Unknown	Unknown	Unknown	Pre-clinic
			Medical Device [131]						
SceneRay	Aaxon NDTM [132]	DBS for MD	CE [133]	8 (2x4)	Non-rechargeable battery	Continuous Programmable	No	No (but modular)	Pre-clinic
SceneRay	Aaxon RNDTM [132]	DBS for MD	CE [133]	8 (2x4)	Rechargeable battery	Continuous Programmable	No	No (but modular)	Pre-clinic
Synchron	Stentrode [134]	BCI	IDE [135, 136]	1647	Unknown	No	Yes	Unknown	Pre-Clinic

Alternative neuromodulation strategies aiming to reduce invasiveness, expand indications, or diversify targets and delivery methods within neuromodulation emerge as well. For example, Motif Neurotech’s Dot system explores adaptive TMS-like stimulation for mental health disorders [47]. Precisis GmbH’s EASEE® system offers a minimally invasive, subgaleal stimulator for focal epilepsy, with CE approval and FDA’s Breakthrough designation [48]. Salvia BioElectronics’ MySalvia device targets migraine therapy with an unconventional flexible electrode array implanted subcutaneously [49].

In the field of iBCIs, Blackrock Neurotech and Neuralink are currently among the most prominent and widely recognized companies. Blackrock’s Utah Array has become the research standard for intracortical iBCIs and is FDA 510(k) cleared for investigational use [50]. It supports up to 256 channels and is widely used in academic settings [11]. Neuralink’s Link system features up to 1024 channels, wireless data transmission, and external inductive powering [51]. It is in early human trials under an FDA IDE. Both of these platforms rely on microelectrode arrays, which provide high-resolution signals but at the cost of surgical invasiveness and long-term viability due to tissue response [10, 11]. In contrast, alternative approaches using macroscale electrodes offer less spatial resolution but improved safety and stability. For example, Onward Medical® WIMAGINE uses epidural electrodes, and combines them with SCS to support motor function recovery [42]. Synchron is developing a minimally invasive endovascular system that delivers electrodes via the vasculature, avoiding open-brain surgery entirely. Beyond the major players, a large ecosystem of startups and academic spin-offs is actively developing new platforms, often supported by FDA’s Breakthrough designations and investigational studies.

The field is fueled by ongoing innovation in novel materials, miniaturization, and biocompatibility. Emerging advances in thin-film electrode design and high-density recording interfaces may further enhance the fidelity and scalability of future translational systems (see section 3.2. in supplement). At the same time, novel adaptive algorithms are expanding the functional potential of these devices. This dynamic environment reflects a rapidly evolving field that still has to navigate challenges in scalability, economic viability, and regulatory pathways.

### 2.2. Clinically approved adaptive neurostimulation

#### 2.2.1. Neuropace RNS

The NeuroPace RNS® system is the first FDA-approved RNS device, marking a milestone in closed-loop neuromodulation. It is approved for the treatment of drug-resistant epilepsy [52]. The system continuously monitors brain activity and delivers targeted stimulation when seizure patterns are detected, aiming to disrupt seizure initiation while avoiding unnecessary stimulation [23]. This approach has shown long-term benefits in seizure reduction [53] and exemplifies the potential of adaptive real-time neuromodulation in clinical applications.

#### 2.2.2. Medtronic BrainSense technology

Recently, the FDA approved Medtronic’s BrainSense^TM^ technology, making it the first commercial closed-loop DBS system approved for treatment of *Parkinson’s disease, essential tremor,* and *dystonia* [54]. The system consists of Percept^TM^ neurostimulators, BrainSense^TM^ software, and SenSight^TM^ directional leads [55]. Unlike conventional continuous DBS, this system adjusts stimulation based on real-time neural activity, improving symptom management in movement disorders while reducing side effects and power consumption [16, 26]. The approval reflects ongoing progress in adaptive neuromodulation and highlights the growing interest in personalized, responsive stimulation strategies.

### 2.3. Translational platforms and ecosystems

A major challenge in both iBCIs and neuromodulation is understanding of how neurological disorders and brain stimulation affect neural circuitry. While progress has been made, a full mechanistic understanding is still lacking [19]. Gaining this insight could enable more effective therapies and wider clinical adoption [2, 10, 56].

One key reason for the knowledge gap is the complexity of the stimulation parameter space [57], which includes frequency, amplitude, timing, and spatial targeting, along with the limited availability of research subjects, especially those with chronic implants and long-term data [11]. Much of the existing data remains siloed within institutions or companies, with access often restricted by privacy regulations or intellectual property concerns [58]. These barriers continue to impede the development of generalized models and scalable therapeutic strategies.

To accelerate the development of these therapies, we need a rapid, systematic hypothesis experimentation that maximizes every opportunity to collect and interpret neural data. This calls for the development and deployment of **translational platforms**—experimental ecosystems that bridge preclinical and clinical environments, enabling iterative development, real-time testing, and refinement of neuromodulation strategies [2, 10, 13, 15].

Rather than building entirely new systems, greater value can be achieved by upgrading existing technologies through hardware and firmware updates. This highlights a key design requirement: **translational platforms should be modular in both hardware and software**. Modularity allows new therapies to be retrofitted into existing systems, whether through firmware updates or external software layers. It also enables the seamless integration of experimental features into clinical workflows without needing full device replacement. This flexibility is crucial for reducing cost, accelerating clinical deployment, and maintaining continuity of care.

**Figure 1.**
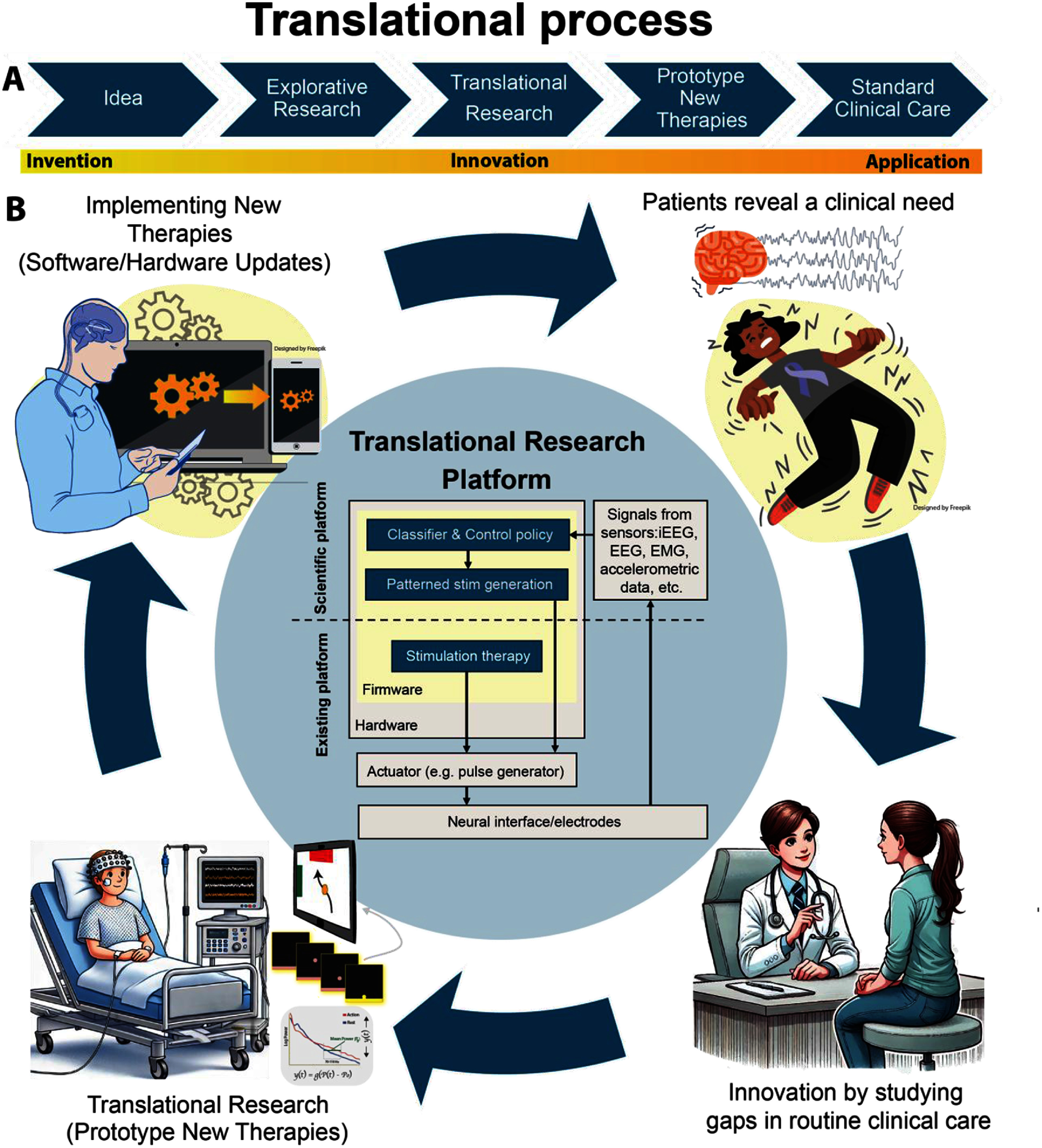
Illustration of translational process for neuromodulation therapies. (A) Translating an idea into standard clinical care requires ensuring that an invention evolves into a practical application. (B) A model of translational research and development may be enabled using a flexible neuromodulation research platform. New therapies are driven by clinical needs and the gaps in current practice. By addressing these gaps, prototypes of novel therapies can be developed and refined in an iterative process. Once proven effective and safe, these therapies can be disseminated within the research platform or integrated into contemporary devices via software and hardware updates. Reproduced with permission from [15]. © 2020 Elsevier Inc. CC BY-NC 4.0.

A translational platform could be exemplified by the following scenario (figure 1): A patient with drug-resistant epilepsy is admitted to an EMU for seizure localization. During this time, the patient consents to participate in research to improve epilepsy care and explore biomarkers for related conditions such as mood disorders [59]. After evaluation, the patient qualifies for an INS. Together with the clinical team, they choose a responsive, closed-loop device with recording capabilities, supporting long-term data collection beyond the hospital setting. This setup enables the system to optimize therapy while studying additional neural signals, utilizing the full potential of the implanted device. If the patient later develops major depressive disorder (a common comorbidity of epilepsy [59]) and medications are ineffective, the clinical team may reprogram the existing device to deliver targeted stimulation during symptom elevation episodes. At the end of the device’s life, the patient may opt for an upgraded version supporting more electrodes or improved sensing modalities. Due to its modular design, much of the implanted system, such as leads, can be preserved, minimizing surgical burden and expanding capability. While translational platforms offer significant promise for accelerating neuromodulation development, they also introduce complex **regulatory, legal, and ownership challenges**, particularly in modular systems composed of components from different developers [14, 56]. Defining intellectual property, regulatory responsibility, and liability is essential, yet unresolved questions remain: *Who owns the data? Who is liable in case of failure? How should modular components be regulated?* In such cases, traditional regulatory pathways may not apply. Responsibility may fall to clinicians, institutions, or third-party integrators submitting new regulatory packages. Though the FDA permits modular PMA submissions, they typically involve a single sponsor. Reimbursement frameworks are similarly unclear; CED through CMS may be required to establish billing codes, potentially setting regulatory and reimbursement precedents for future modular neuromodulation systems.

An additional layer of complexity arises in closed-loop systems, which depend on reliable, real-time biomarkers. Yet, biomarker design is complicated by signal instability, symptom variability, and the limited temporal scope of studies, often not fully capturing brain temporal dynamics and internal states like mood or hormonal cycles [60–62]. To ensure safety and adaptability, more emphasis should be also placed on incorporating a **fallback mode** (a predefined safety state activated in case of malfunction), as well as **defining safe stimulation zones** and enabling continuous monitoring of both patient and device states with alert systems.

These challenges are complex, but as early adopters implement these systems, their experience will guide best practices and inform evolving regulatory frameworks. Their efforts will be key to enabling safe, compliant, and collaborative innovation in neuromodulation.

#### 2.3.1. CorTec’s BIC platform

**Figure 2.**
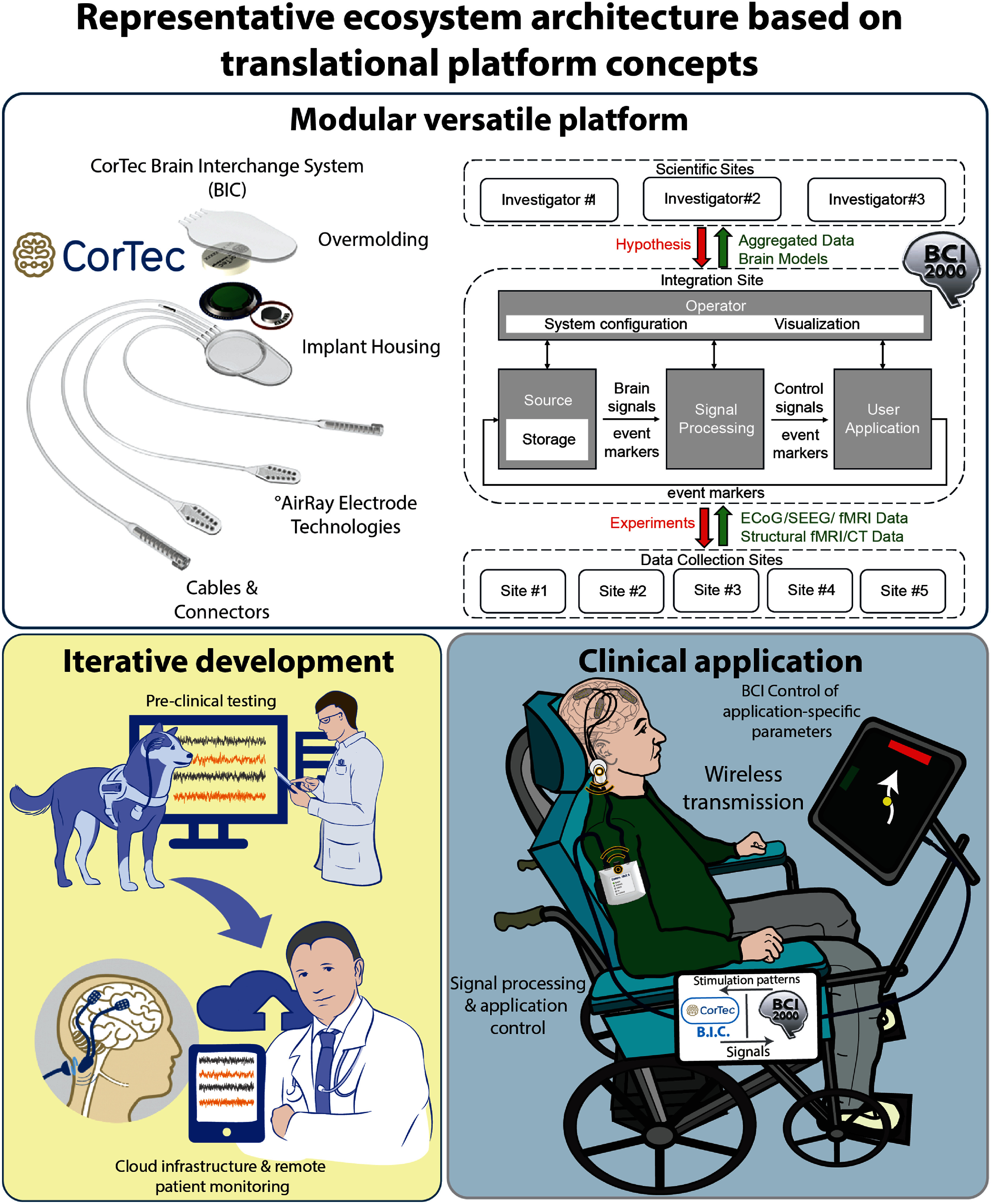
Representative ecosystem architecture based on translational platform concepts. **Top panel**: A versatile modular platform ecosystem consisting of the Brain Interchange System (BIC) and BCI2000 software. The left side shows the BIC implant and its components; the right side illustrates the work-flow and implementation of BCI2000 in multi-center studies. Adapted with permission from CorTec. **Bottom left**: Iterative development in preclinical settings using canine models to develop a robust platform for therapeutic applications, including support for in-home use and cloud-based synchronization. Adapted with permission from [64]. Copyright © 2022, Published by Oxford University Press on behalf of the Guarantors of Brain 2022. This work is written by (a) US Government employee(s) and is in the public domain in the US. **Bottom right**: Envisioned therapeutic application of the CorTec BIC–BCI2000 platform to support in-home use for amyotrophic lateral sclerosis (ALS) patients with locked-in syndrome, enabling wireless signal processing and application control.

One of the emerging devices suitable for application in adaptive neuromodulation and iBCIs is CorTec’s BIC (figure 2). It is a fully implantable device, capable of sensing and stimulation [78]. The device is customizable and capable of supporting surface ECoG constructs and DBS leads, with up to 32 channels sampled at 1 kHz. All of the device technical specifications and applications are available at CorTec’s website. The device API is available on different platforms including C/C++, Python, and Matlab, allowing the neurotech community to develop open-source solutions (BCI2000 [63], OMNI-BIC [27]).

#### 2.3.2. Data integration in neural research

Neurotechnology research is an interdisciplinary field that requires a wide range of skills and knowledge. Translating scientific ideas into a real-world clinical application requires collaboration across multiple fields, including neurosurgery, signal processing, and computational engineering. Such broad knowledge necessitates interdisciplinary teams, often geographically dispersed, using heterogeneous hardware, and a variable level of training. These limitations can create situations where a research idea remains stuck in translation, due to the many potential failure points in this process.

A commonly encountered issue is the dependency on proprietary knowledge, such as annotations, naming conventions, or synchronization of various data streams, which is often necessary to analyze datasets effectively. Fluctuations in the personnel holding this knowledge may lead to a loss of the information, and result in the unusability of the whole dataset. To address this issue, several research platforms were proposed to fully document and streamline the data collection process. A non-exhaustive list is provided in table 2. Each platform reflects distinct design priorities and trade-offs [65]. For example, BCI2000 [66], Dareplane [67] and OpenViBE [68] emphasize modularity and accessibility for experimental BCI research; ROS-Neuro extends neural interfacing into the domain of neurorobotics [69]; Lab Streaming Layer (LSL) [70] addresses the critical need for synchronized multimodal data acquisition; FieldTrip offers advanced analytical capabilities for neurophysiological data within MATLAB [71]; while MedusaBCI [72] and NeuroPype [73] provide Python-based and commercial-grade environments for real-time signal processing and integration with modern computational tools. These platforms aim to simplify experimental design and streamline data acquisition. However, once data are collected, new challenges arise,such as how to **standardize, share, and retain usability of datasets across institutions**. These issues are particularly relevant for reproducibility and translational research. We explore them further in section 5.1.6: *Standardization of data acquisition, storing, and sharing.*

**Table 2.** Non-exhaustive list of popular BCI platforms for research and commercial use [65–73]. Reproduced from [67]. © The Author(s). Published by IOP Publishing Ltd. CC BY 4.0.

Platform	Primary programming language	Purpose & design philosophy	Licensing & accessibility	Supported hardware systems
BCI2000	C++	General-purpose modular BCI system for research and clinical use.	Open-source (GNU GPL) [74]	50+ systems
OpenViBE	C++	User-friendly platform for BCI in VR and real-world environments.	Open-source (AGPL) [75]	20+ systems, plug-in extensible 12 systems,
ROS-Neuro	C++	Middleware for neuroscience and robotics integration.	Open-source (MIT License) [76]	LSL compatible
Lab Streaming Layer (LSL)	C++	Middleware for synchronized, real-time data streaming.	Open-source (MIT License) [70]	90+ systems, via community plug-ins
FieldTrip	Matlab	MATLAB toolbox for offline/real-time neuro data analysis.	Open-source (CC BY-NC 2.0) [72])	Compatible via buffer streaming (e.g. BCI2000)
MedusaBCI	Python	Python-based BCI platform with modular design.	Open-source (GNU GPL) [77]	LSL-compatible systems
NeuroPype	Python	Comprehensive real-time BCI and neuroimaging suite.	Commercial (free academic edition) [73]	LSL-compatible systems
Dareplane	Python	Minimalistic, low-latency modular BCI software ecosystem.	Open-source (MIT License) [67]	LSL-compatible systems

#### 2.3.3. BCI2000

BCI2000 provides a free-to-use ecosystem that streamlines collaboration across institutions and maintains coherence through generations of scientists [66]. BCI2000 stands out for its versatility and broad adoption [65]. It is a general-purpose, open-source platform provided and maintained by NCAN. BCI2000 supports over fifty different hardware systems [79], and is designed to unify and integrate the entire data collection process. Built in C++, it offers high performance for real-time data acquisition and processing. In addition, it also supports third-party application extensions and provides interfaces to both MATLAB and Python, enabling seamless integration with widely used analysis environments and facilitating the development of custom processing pipelines for complex research projects (figure 2). Maintaining the data coherence across time is ensured by BCI2000’s native data format, which records and documents all details and events occurring during an experiment. Moreover, BCI2000’s design ensures that the experiment is properly set up before allowing a user to perform the experiment.

#### 2.3.4. Other examples of translational platforms

The concept of iterative development is becoming increasingly established in neuromodulation and iBCIs research. Several companies are actively building translational platforms to prototype, test, and refine therapies across preclinical and clinical settings. Medtronic’s Summit RC+S is an investigational device combining sensing and stimulation for prototyping closed-loop neuromodulation therapies [80]. It is used in research on PD [81–83], epilepsy [84] and psychiatric disorders [31, 82]; however, the device has been discontinued by the manufacturer. The accumulated knowledge contributed to the development of BrainSense^TM^ technology, the sensing technology now used in commercial DBS systems. Cadence Neuroscience is an emerging company developing a clinical platform that includes a bedside workstation and a permanent 4-lead INSR device [85]. This system allows clinicians to test and optimize biomarker-targeted stimulation in real time during inpatient monitoring (e.g. in the EMU) [86]. Once optimized, therapy continues with the implanted device, which enables ongoing seizure monitoring and adaptive adjustments [86]. Ripple Neuro offers modular, high-channel-count platforms for preclinical research, supporting closed-loop recording and stimulation in animals. Its flexible architecture allows researchers to combine processors, front-ends, and I/O modules to match experimental needs. Building on the expertise, Iris Biomedical’s Athena (a Ripple venture company) was created to develop clinical-grade implantable systems. Its platforms include two systems: ATHENA, a long-term implantable system with programmable stimulation, multi-modal sensing, and wireless power/data transfer; and the Pilot Handheld, a portable, 256-channel system for real-time neurophysiology processing and rapid algorithm testing [87]. Translational platform development is also advancing in the software domain, where tools are designed for rapid exploration of stimulation parameters. These platforms are often modular, open-source, and support iterative therapy development. Examples of such platforms include *Boston Scientific’s Chronos* research software, which leverages the flexibility of the commercially available Vercise Genus*^TM^* stimulators by enabling flexible exploration of stimulation parameters within established safety limits [20, 88]; the *Dareplane neuroplatform*, a modular open-source environment developed for neuromodulation research with a focus on aDBS [67]; and the *OpenMind Consortium* and its associated tools created by collaborative efforts [82].

## 3. Clinical need

Limitations in conventional treatments drive the development of new clinical neuromodulation therapies. To justify their full translation into clinical practice, new neuromodulation therapies must offer clear advantages over the status quo, such as improved efficacy, lower costs, enhanced safety (e.g., less invasiveness), or better patient accessibility. However, successful translation into standard clinical practice requires a balanced approach; a therapy that enhances efficacy but significantly increases costs may not be viable. Economic incentives, together with patient accessibility and usability, play a critical role in determining which tools and treatments become widely adopted [10]. This section provides an overview of current clinically oriented research in neuromodulation and its implications for the future of neuromodulatory therapies.

### 3.1. Translational pathways for current and future therapies

#### 3.1.1. iBCI solutions for ALS induced motor loss

ALS is a progressive, fatal paralytic disease due to neurodegeneration of motor neurons that reside within the frontal cortex of the brain and anterior horn of the spinal cord [137]. The average duration of symptom onset (muscle weakness, difficulty chewing or swallowing) to death due to respiratory failure is 3–5 years [138]. There is considerable variability in clinical phenotypes and rates of progression. While considerable effort is ongoing to develop new medications that slow disease progression, there is a significant unmet need for therapies that improve quality of life [139]. Recent developments in ECoG-based iBCIs (figure 2) aim to address this need by enabling enhanced communication, environmental control, and autonomy/agency for patients with ALS. Importantly, this provides people living with ALS improved quality of life within their homes, which they can utilize on a daily basis. The goal is to develop a iBCIs that: (1) allows patients to learn the system quickly, (2) works with existing communication and environmental control tools, (3) is easy to use both at home and in external environments, and (4) requires minimal technical support. While there is growing interest in iBCIs applications within the ALS community, several technological and clinical challenges persist, namely: (1) the type of iBCIs that may help someone early in the disease may be different than those late in the disease, (2) speech and language is a distinct problem from inability to use one’s arms and legs, and (3) defining clinical endpoints that will satisfy patients & their communities alongside regulators. Finally, it is still unclear how stable a iBCIs signal will remain for individuals suffering from a neurodegenerative process. A recent study suggests that a usable ECoG signal remains over seven years out from implantation [140], but further work remains.

#### 3.1.2. Protocols for adaptive neuromodulation: from animal models to research in humans

Current iBCIs systems require a high level of expertise and substantial infrastructure to operate effectively. To address these limitations, a robust open-source iBCIs ecosystem is being developed to support novel therapies for patients with neurological disorders. This ecosystem consists of the CorTec’s BIC implantable device (section 2.3.1) and the BCI2000 software platform (section 2.3.3). While the current implementation integrates specific hardware, the platform has been designed with portability in mind and can be adapted to other systems, following the principles of translational platform development [63]. The development of such versatile and robust ecosystem necessitates thorough verification of the system’s capabilities through rigorous testing and iterative troubleshooting [10, 63]. In this context, preclinical validation of the BIC-BCI2000 ecosystem is being conducted in canine models (figure 2). Canines are not commonly used in neuromodulation research; however, they offer several advantages, including neurophysiological and pathophysiological similarities to humans, cooperativity, and domesticated social behavior. Moreover, all dogs are made available for adoption upon completion of the study^22^22Canines are put up for adoption upon the study’s completion in compliance with the State of Minnesota statute 135A.191., allowing them to lead fulfilling lives beyond their role in research. To support this work, specialized surgical procedures were developed to accommodate the anatomical and ethical considerations specific to canines [63]. System was continuously evaluated for longevity and functionality, by daily recordings. Approximately one year post-implantation, a series of functional experiments were conducted involving sensory, visual, and social tasks. These experiments led to unexpected neurophysiological findings related to social processing in the canine brain, contributing to a deeper understanding of the evolution of social capabilities in mammals [141, 142].

While the BIC-BCI2000 ecosystem is being developed to support various therapeutic applications, the long-term goal is to enable in-home use for individuals with neurodegenerative conditions such as ALS who experience locked-in syndrome. Broadband spectral changes recorded from implanted electrodes have been shown to be effective for iBCIs applications [143], and recent work by Jensen *et al* [144] demonstrates the feasibility of using these neural signals for device control. Building on this foundation, BIC-BCI2000 ecosystem is designed to integrate with existing assistive technologies (e.g. Wego^TM^ or Tobii) during the early stages of the disease when motor function remains. As motor abilities decline, the ecosystem would gradually assume BCI-based control, allowing patients to continue communicating and interacting with their environment using only brain activity, such as controlling a virtual keyboard or mouse through broadband signal modulation.

#### 3.1.3. Neuromodulation therapies in movement disorders

Current DBS therapies primarily rely on continuous open-loop stimulation, in which stimulation is delivered at fixed parameters regardless of ongoing brain activity [16, 19, 145]. Many widely used systems, such as *Abbott Infinity* and the *Boston Scientific Vercise Gevia family*, operate without sensing capabilities, providing clinicians only limited opportunities, typically during implantation or battery replacement, to observe neural signals and adjust the stimulation parameters accordingly. As a result, parameter adjustments are made gradually (and often slowly) over time based on patient feedback [2]. While this approach has proven effective in reducing symptoms in movement disorders [16, 26, 145–148], its lack of responsiveness to dynamic behavioral or environmental states can lead to suboptimal outcomes and side effects, including impaired proprioception, gait disturbances, and speech dysfunction [16, 26]. aDBS seeks to overcome these limitations by adjusting stimulation in response to real-time physiological or behavioral feedback [16, 26, 147, 148]. Its potential has been demonstrated in several studies, and comprehensive reviews by Neumann [148], Priori [147], and Lozano [145] outline the advances in these therapies. The first commercial approval of a closed-loop DBS system for movement disorders marked a major milestone, achieved with FDA’s approval of *Medtronic’s BrainSense technology* (see section 2.2.2), which is integrated into their Activa PC + S system. Prior to this, several studies explored on-demand DBS paradigms, which aim to optimize therapy by tailoring stimulation to behavioral intent. One such example involved decoding upper limb movement intent from cortical surface recordings and triggering thalamic stimulation upon detection [26]. This approach achieved comparable efficacy to standard continuous stimulation while reducing energy consumption by *∼*52% and minimizing stimulation-related side effects [26]. Moreover, it enabled semi-automated training of stimulation parameters in the spectral domain, paving the way for smoother integration into clinical practice [26].

#### 3.1.4. Brain state modeling for adaptive closed-loop neuromodulation in epilepsy

Progress in the development of effective closed-loop neuromodulation systems for various neurological disorders remains limited by the challenge of accurately characterizing the brain’s instantaneous functional state. Traditional approaches often rely on expert-labeled EEG data to define biomarkers of neurophysiological pathology. However, these methods fall short in capturing the full complexity of brain dynamics, particularly the transitional or intermediate states that lie between labeled conditions, as well as the variability that may exist among identically labeled states [149]. To address these limitations, *BrainState*, a self-supervised framework, has been introduced to model arbitrarily complex, moment-to-moment brain states using multivariate neural time-series data. Applied to iEEG recordings from patients with epilepsy in EMU, *Brain-State* demonstrated the ability to identify a rich spectrum of preseizure states and quantify nuanced effects of neuromodulation [149]. The long-term goal of this approach is to facilitate the creation of advanced closed-loop neuromodulation systems tailored to a broad range of neurological conditions.

#### 3.1.5. A next generation epilepsy management ecosystem–integrating brain implants, wearables & cloud infrastructure

Epilepsy affects millions of people worldwide, and its unpredictable nature significantly impacts quality of life [150]. Current clinical management relies heavily on patient self-reporting and limited in clinic observations, leading to inaccurate seizure assessment and suboptimal treatment strategies. RNS and DBS devices offer a promising approach [151, 152], but existing systems have limitations in sensing capabilities, stimulation paradigms, and real-time patient feedback. While various tools and approaches for adaptive closed-loop neuromodulation in epilepsy have been explored, as comprehensively reviewed by Rao and Rolston [153] and Starnes *et al* [154], the only FDA-approved RNS system remains NeuroPace RNS (section 2.2.1) [52].

**Figure 3.**
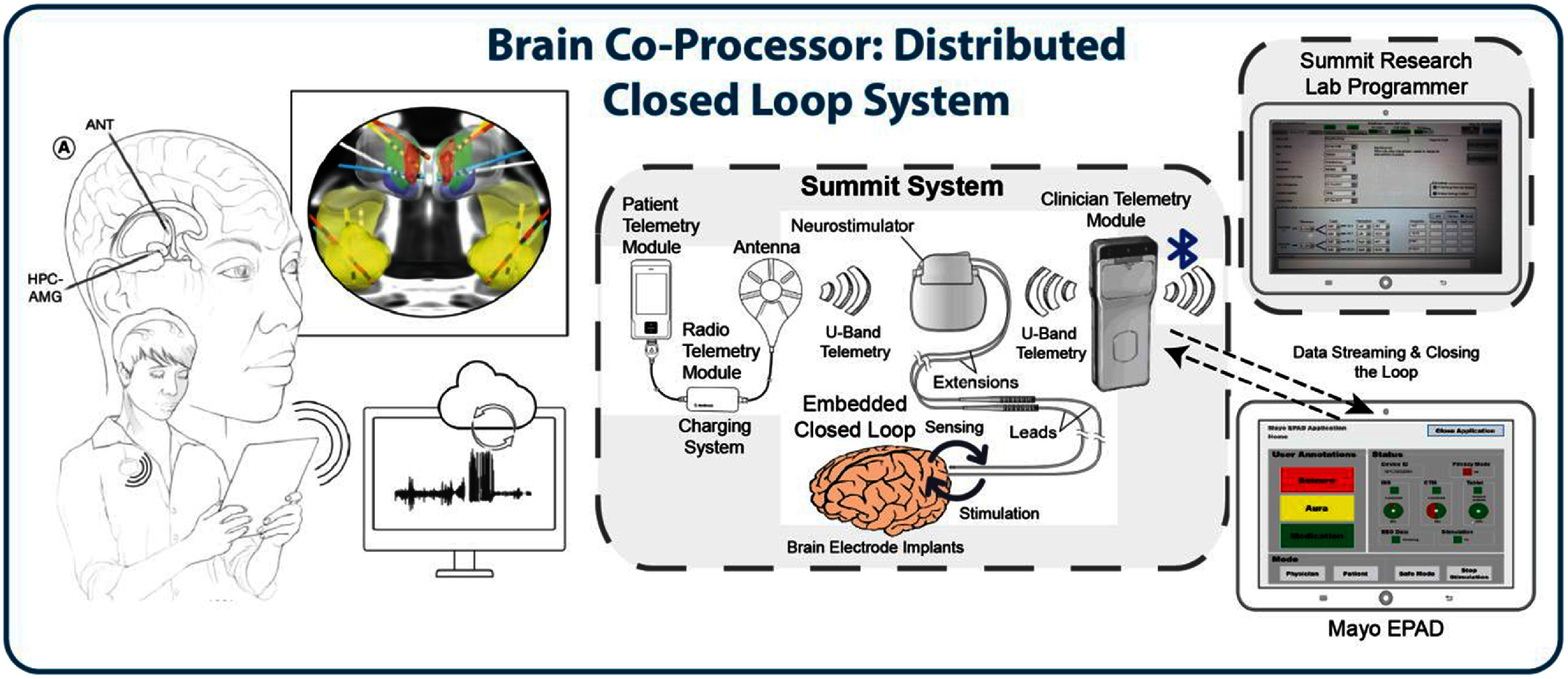
Brain Co-Processor: System for brain sensing and stimulation in epilepsy, integrating wireless data streaming and communication with the phone and wearable devices. **Left**) Schema of implantation targets in anterior nucleus of thalamus (ANT), hippocampus (HPC) & amygdala (AMG) together with brain segmentation images with corregistered electrodes implanted in five patients. The data are streamed continuously to the mobile device and transferred to the cloud when the internet is available. **Right**) The schema of the Medtronic’s Summit RC+S system [80], consisting of the implantable neural stimulator and peripheral hardware, including patient charger and controller, U-Band & Bluetooth telemetry, and patient mobile device, alongside the physician programmer that is used to program safety limits and configuration of electrodes and programs. Reprinted, with permission, from [84]. © [2018] IEEE.

An alternative approach has been developed through the *Mayo Clinic BrainRISE platform*—a novel device-agnostic ecosystem. It integrates data from an implantable RNS device with a secure cloud infrastructure, enabling continuous streaming, analysis, and adaptive stimulation. Designed to support multiple modalities, *BrainRISE* allows researchers to aggregate data from implants, wearables, and mobile devices, offering a flexible alternative to closed, single-vendor systems. The core of the ecosystem (figure 3.) is the investigational Medtronic Summit RC+S device [80], capable of multi-target sensing and stimulation, programmable detection algorithms, and continuous data streaming. Initially validated in epileptic canines [84], it was later implanted in five patients with medically refractory epilepsy, targeting bilateral hippocampi and ANT [64]. The device features dual embedded detectors enabling closed-loop paradigms, including targeted stimulation within the hippocampus and adaptive adjustments across stimulation sites or parameters. Additionally, real-time seizure-triggered questionnaires delivered via smartwatch or smartphone provide patient-reported outcomes to inform therapy optimization [64]. A custom-designed technology stack was developed to interface with the INS, comprising:

• **Wearable Interfaces**: A smartwatch and smartphone application provided patients with real-time feedback on device status, medication reminders, and event logging capabilities.
• **Cloud platform with advanced analytics and visualization tools**: Continuous data from the INS was streamed to a tablet and securely relayed to a HIPAA-compliant cloud platform. All data were encrypted and deidentified. Cloud-based infrastructure enabled sophisticated data analysis, including seizure detection, spike rate quantification, automated sleep scoring [155], and biomarker identification. Physicians could access this information through a user-friendly interface to monitor patient progress and adjust treatment strategies remotely.

Preliminary data from five implanted patients have shown the feasibility of continuous data streaming and closed-loop stimulation. The system achieved a high data capture rate (70% on average), enabling detailed analysis of brain activity and seizure patterns [64]. By integrating continuous brain sensing, adaptive stimulation, and real-time patient feedback, this novel ecosystem provides a comprehensive platform for personalized, data-driven neuromodulation therapies. Future developments will focus on refining algorithms, integrating multimodal data, expanding applications to other neurological disorders, and adapting the ecosystem to new platforms following the discontinuation of the Medtronic RC+S system [156].

#### 3.1.6. Early feasibility iEEG recordings from human subjects using the CorTec’s BIC syste

Neurosurgical interventions can provide an effective alternative in the treatment of patients with pharmacoresistant epilepsy [157, 158]. However, the success of the interventional surgical procedures largely depends on the accurate identification of the SOZ [159]. HFOs in iEEG recordings have emerged as promising clinical biomarkers for identifying epileptogenic brain regions [160]. Characterized by brief, synchronized transients in the 80–500 Hz range [161], their detection has improved through time-frequency analysis [162] and sparse representation methods, differentiating between true HFOs and pseudo-events [163–165]. However, existing clinical implantable devices are limited by their low number of acquisition channels (typically 8–16 contacts), battery constraints and low sampling rates (up to 256 Hz), that restrict them for monitoring the high-frequency neural events on the long-term basis (see table 3) [42, 63, 113, 166–169].

**Table 3.** Comparison of representative implantable neural stimulator systems. The table compares key features of three DBS devices (Medtronic Percept, Abbott Infinity, and Boston Scientific Vercise), the NeuroPace RNS system, the WIMAGINE research platform, and the CorTec Brain Interchange (BIC) system [42, 113, 166–169]. Adapted from [169]. CC BY 4.0.

System	Recording	Sampling rate	No. channels	Stimulation	Max. stimulation frequency	Max. stimulation current	Max. pulse width
Percept	✓	250 Hz	16 (2 × 8)	✓	250 Hz	25.5 mA	450 *µ*s
Infinity	×	N/A	16 (2 × 8)	✓	240 Hz	12.75 mA	500 *µ*s
Vercise	×	N/A	16 (2 × 8)	✓	250 Hz	12.7 mA	450 *µ*s
RNS-320	✓	250 Hz	8 (2 × 4)	✓	333 Hz	10 mA	1000 *µ*s
WIMAGINE	✓	32Ch (1 KHz) 64Ch (600 Hz)	64	×	N/A	N/A	N/A
BIC	✓	1000 Hz	32	✓	200 Hz	6.12 mA	2500 *µ*s

BIC system is a next-generation wireless, implantable and externally powered device, capable of high-resolution neural data acquisition (32 contacts at 1000 Hz) and responsive stimulation. The BIC system addresses this critical need in the neuromodulation field by enabling data acquisition at more brain sites and at high sampling rates to evaluate high and low frequency neuro biomarkers (HFOs/interictal spikes) of epilepsy [169, 170].

The feasibility of the BIC system was evaluated in a two-phase study using iEEG recordings from patients in EMU. In the first phase, 24-hour iEEG recordings were acquired from three patients, and the signal quality was compared against FDA-approved clinical systems *Nihon Kohden* and *Natus Quantum® (NQ)*. Evaluations included raw data correlation, time–frequency analysis, power spectral density, noise density, and spatiotemporal analysis of interictal spike rates [169]. A validated spike detector [171] was applied to both systems using identical parameters. The second phase involved a fourth subject, in whom simultaneous iEEG recordings were acquired using both the BIC and NQ systems [170].

**Figure 4.**
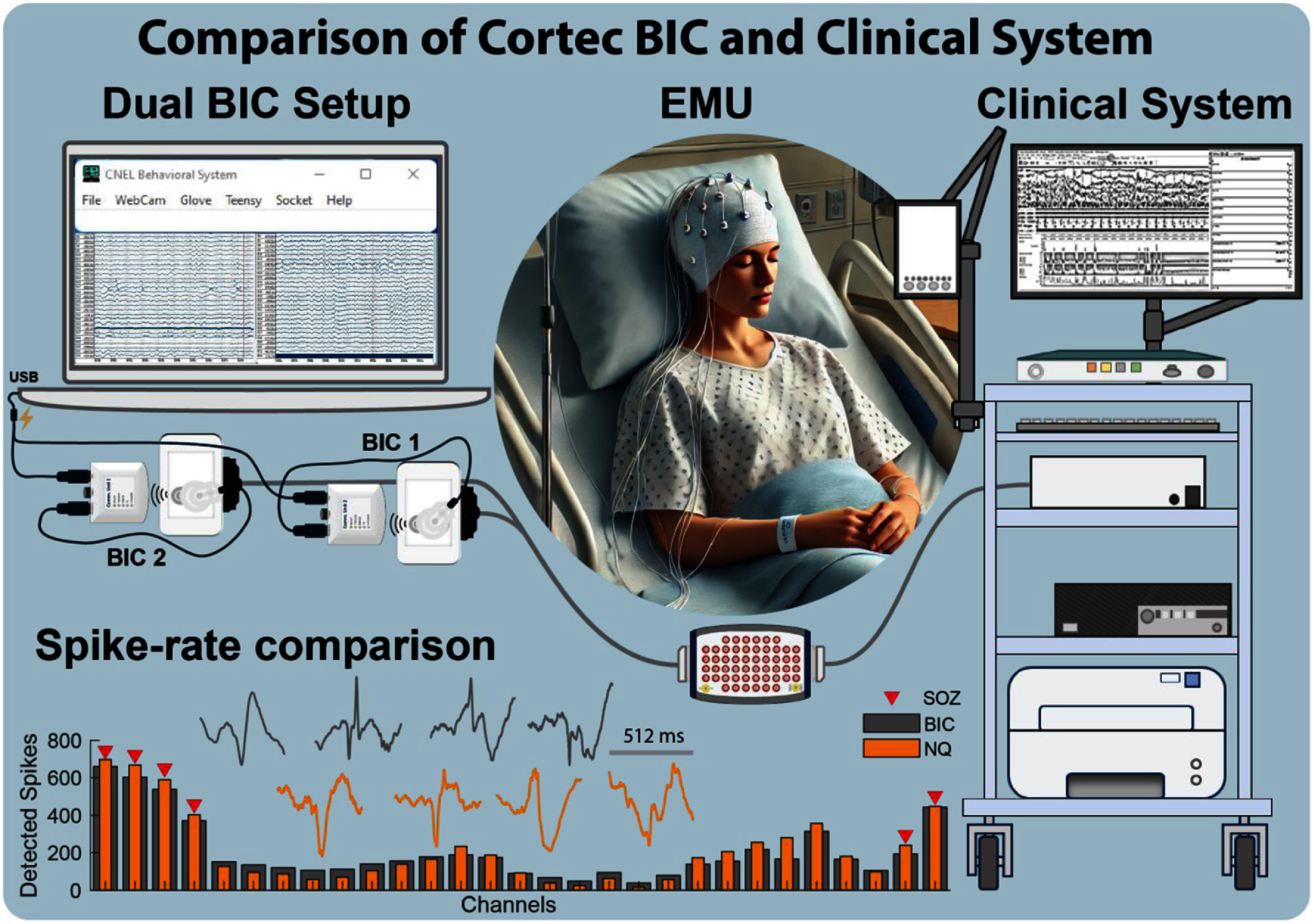
BIC system early feasibility study in human subjects. The CorTec’s BIC benchtop system, designed for prototyping neuromodulation therapies, was evaluated in the epilepsy monitoring unit (EMU) during stereoelectroencephalography (SEEG) recordings for seizure onset zone (SOZ) localization. Recordings from the BIC system were compared to those obtained using the clinical standard Natus Quantum® (NQ) system. Interictal spike detection results from the BIC were comparable to those from the NQ system, marking the potential of BIC as a viable platform for therapeutic use in patients with epilepsy [169]. Adapted from [169]. CC BY 4.0.

Analysis revealed comparable power spectral density below 100 Hz across systems, but higher noise floors in the BIC system at frequencies where HFOs typically occur. Despite this, both systems recorded similar rates of interictal spikes with closely matching spatial and temporal distributions (figure 4). High-frequency signals were found to be more susceptible to data packet loss, particularly above 100 Hz [169, 172]. To mitigate this issue, the team implemented solutions to manage electromagnetic interference and reduce packet loss, including firmware-based triggers that automatically initiate hardware restarts when packet loss exceeds a defined threshold [169].

Future development aims to integrate newly developed real-time interictal spike [173] and HFOs [174] detection algorithms with the BCI2000 platform [66], enabling portable, device-agnostic systems capable of real-time data streaming, analysis, and feedback.

## 4. Neuroscientific understanding

The first DBS systems emerged in the 1980s with the aim of treating symptoms of PD and essential tremor [2]. More than 40 years later, the precise mechanisms underlying these therapies remain only partially understood [175]. Despite this knowledge gap, neuromodulation therapies have demonstrated effectiveness empirically, improving symptoms across a range of neurological conditions. Depending on the targeted brain region and interfacing strategy, invasive therapeutics can serve multiple functions:

• **Inactivation of brain regions or adjacent circuitries** through high-frequency stimulation, as seen in DBS (*∼*130 Hz [18, 19, 176]) and SCS (up to 10 kHz [177, 178]).
• **Modulation of neural circuits through mid-frequency stimulation** (*∼*60 up to 100 Hz), which can drive oscillatory brain activity [19, 176]. For example, low-frequency DBS has been explored for treating bradykinesia in PD and for enhancing neuroplasticity in motor recovery [179].
• **Detection and interruption of pathological activity** through RNS, where stimulation is delivered upon detecting abnormal neural patterns, such as in epilepsy management [151, 152].
• **Decoding of intention** and improving life quality through various iBCIs applications for paralyzed patients [42, 43, 139].
• **Paired decoding and stimulation to induce plasticity** and rehabilitate neural tissue, such as in post-stroke recovery [28, 180, 181].

Additionally, iEEG provides direct insights into brain electrophysiology, offering a valuable tool for understanding both normal and pathological brain function. This knowledge has been applied in several clinical areas, including surgical planning, pharmacokinetic analysis, and biomarker development for guiding future therapies.

### 4.1. Functional domains that guide therapeutic interventions in neurostimulation

#### 4.1.1. Dose adjustment in neuromodulation

**Figure 5.**
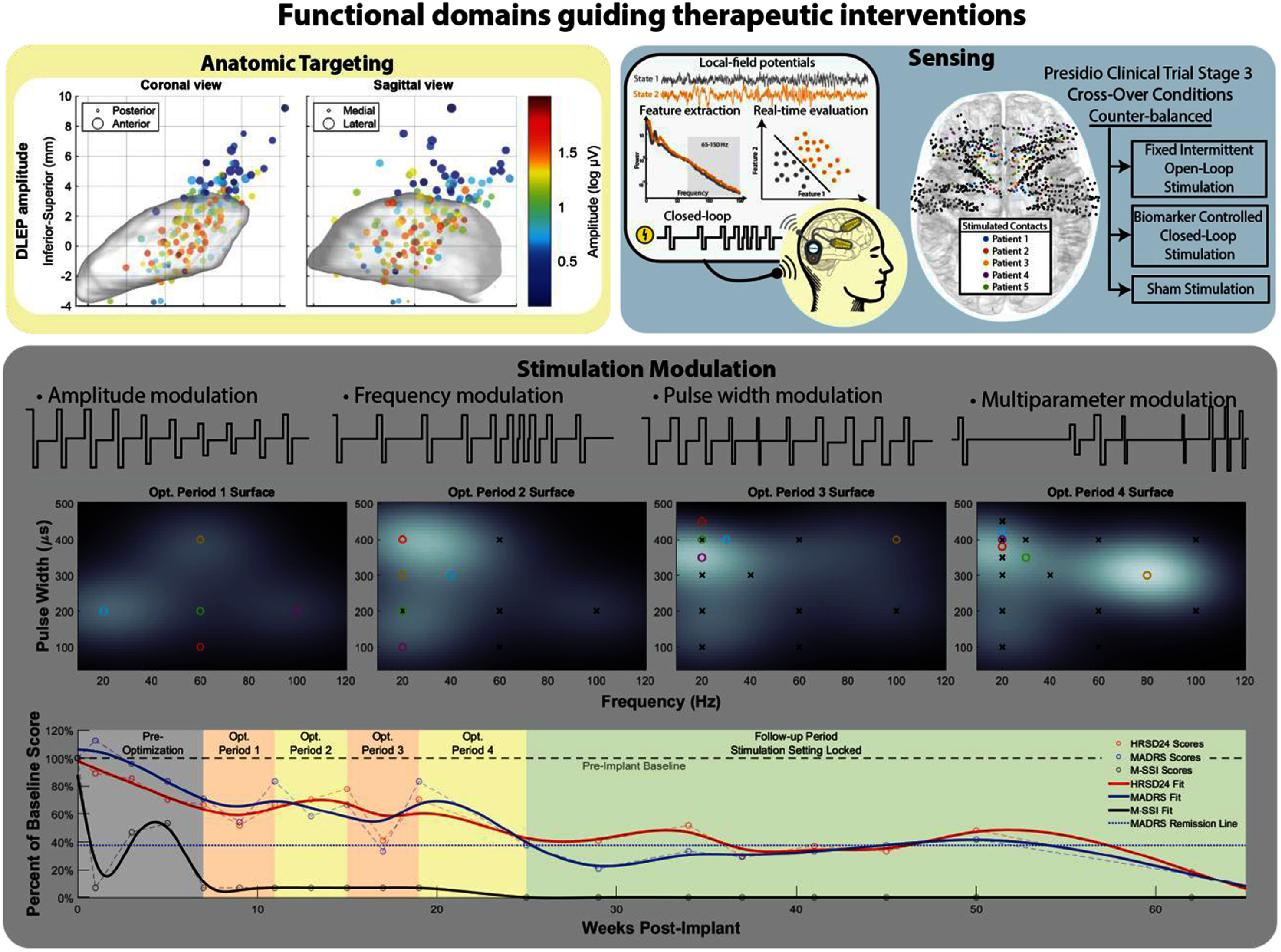
Three main functional domains guiding therapeutic interventions in neuromodulation. **Targeting**: Delivers therapy to specific brain regions. Panel shows the amplitude of the estimated DBS local evoked potentials (DLEPs) [190] mapped to the subthalamic nucleus (STN) center in MNI space (ICBM 152 Nonlinear 2009 [191, 192]), across all patients. Reproduced from [190]. © The Author(s). Published by IOP Publishing Ltd. CC BY 4.0. **Sensing:** Monitors brain states and decodes physiological signals to guide stimulation. *Left*: Schematic of the closed-loop stimulation. *Right:* Presidio clinical trial [32] Stage 1–stereoelectroencephalography (SEEG) electrodes were implanted in five patients to identify personalized biomarkers and stimulation targets in individuals with major depressive disorder. Figure shows color-coded electrode contacts (in superior view) tested for therapeutic stimulation warped to MNI space (ICBM 152 Nonlinear 2009 [191, 192]). **Stimulation modulation:** Current implantable neural stimulators allow for tuning of multiple stimulation parameters, requiring exploration of a large parameter space for patient-specific optimization. Bayesian optimization helps identify optimal settings by iteratively testing new parameters (colored circles) based on prior feedback (black X’s), aiming to maximize therapeutic benefit. The graph shows patient symptom scores over time, measured using Hamilton Rating Scale for Depression (HRSD-24), Montgomery-Åsberg Depression Rating Scale (MADRS), and a modified suicidal severity index (M-SSI). A horizontal line marks the 40% threshold for clinical remission. While scores initially fluctuate as parameters are tested, they stabilize after *∼*25 weeks post-op, indicating convergence on an effective stimulation setting that sustains remission. Reproduced from [193]. CC BY 4.0.

Neuromodulation technologies can be broadly categorized into three functional domains that guide therapeutic interventions (figure 5). The first, **targeting**, focuses on delivering therapy to specific neural regions and is the most extensively studied area, forming the foundation for many established approaches. The second, **sensing**, monitors neuronal states and decodes physiological signals to determine the appropriate stimulation. This domain represents a currently rapidly developing area of research. The third, **stimulation modulation**, designs and encodes stimulation pulse parameters, such as amplitude, frequency, and pulse width, to deliver therapeutic signals that selectively activate target neural elements. This promising, yet underexplored domain, seeks to optimize pulse modulation to enhance both the effectiveness and durability of outcomes. Emerging studies in neuromodulation underscore the transformative potential of stimulation encoding by demonstrating its ability to prevent habituation [182], enhance neural selectivity [183], improve energy efficiency [184], and induce neural plasticity [185]. These findings highlight the possibility that carefully designed, time-varying stimulation patterns could achieve therapeutic benefits extending beyond the active application of therapy. To support this research direction, and thereby accelerate innovation in stimulation encoding strategies, specialized software platforms such as the *Chronos research engine* have been developed [20]. Recent investigations have explored the application of time-varying stimulation patterns in SCS for chronic neuropathic pain (figure 5: Stimulation modulation). Computational modeling has helped optimize stimulation timing, showing improved efficacy in preclinical models [186]. In a rat pain model, modulated stimulation performed comparatevly or better than constant stimulation in behavioral and affective tests [187]. Building on these preclinical advancements, an exploratory clinical study evaluated whether time-encoded stimulation pulses could replicate natural sensory perception in patients undergoing acute SCS [177]. Current efforts focus on a doubleblinded randomized controlled trial evaluating the use of time-varying pulse patterns versus traditional constant stimulation for the treatment of chronic neuropathic pain. The primary objective is to compare the effectiveness of these approaches, while exploratory goals include assessing the durability of therapeutic benefit over time [188, 189].

#### 4.1.2. Optimizing neuromodulation therapies

Modern INS enable the adjustment of multiple parameters, such as frequency, amplitude, and pulse width, creating vast ammount of potential stimulation settings per channel. This immense configurability introduces the challenge of ‘parameter explosion’, which complicates the efficient selection of therapeutic settings. To address this, recent research has employed *Bayesian optimization* as a method to identify individualized stimulation parameters tailored to patient-specific needs and clinical diagnoses [57]. The *Bayesian optimization* is an iterative process, consisting of the following steps:

(i) Fitting an objective function to the data
(ii) Generating an acquisition function
(iii) Finding the maximum of the acquisition function and setting it as the next sampling point
(iv) Augmenting the data and calculating the objective function with the new parameters

In the absence of a standardized objective function for clinical evaluation, some implementations have adopted a patient-guided approach similar to ‘trying on glasses’, where patients test different parameter settings and compare them to the previous configurations (figure 5 Stimulation modulation). Although this patient-centered approach accounts for individual needs and priorities, it also poses challenges in reproducibility and stationarity, as patient conditions and needs evolve over time. Bayesian optimization has been successfully applied in various neuromodulation settings [194], including patients with spinal cord injuries, where optimized stimulation improved leg movement [195], bladder control [196], and sexual function [197]. Additionally, in some cases, SCS induced neuroplasticity, leading to partial restoration of function [198]. Applications have extended to DBS and cortical stimulation, treating conditions such as chronic pain [199], depression [193, 200], and epilepsy [201, 202], showcasing the algorithm’s robustness and adaptability across a range of neuromodulation applications.

### 4.2. Electrophysiological biomarkers research in neuromodulation

#### 4.2.1. Neural biomarkers for DBS in Parkinson disease

DBS of the STN is an established medical treatment of the motor symptoms for PD [21, 22]. A crucial aspect of therapeutic success is accurate localization and implantation of DBS electrodes to the motor territory of the STN. Studies have shown that LFPs can help identify optimal contacts for stimulation in the immediate postoperative period [203]. Moreover, LFPs recordings proved to be stable even over a prolonged time in chronically implanted DBS systems [204] and showed to be a feasible biomarker for modulation of *β* band activity (13–32 Hz) by stimulation, with the attenuation being specific to the stimulation site. These insights have led to the development of a real-time framework for intraoperative analysis of LFPs from micro-electrode recordings to guide electrode placement based on the optimal site of stimulation [21]. Furthermore, LFPs offer insight into PD pathophysiology, revealing distinct electrophysiological signatures in the STN across two major PD phenotypes: *tremor-dominant* and *postural instability/gait disturbance* [205]. These two major clinical phenotypes exhibit distinct electrophysiological signatures within the STN, suggesting that stimulation parameters may be tailored to individual symptom profiles [205].

**Figure 6.**
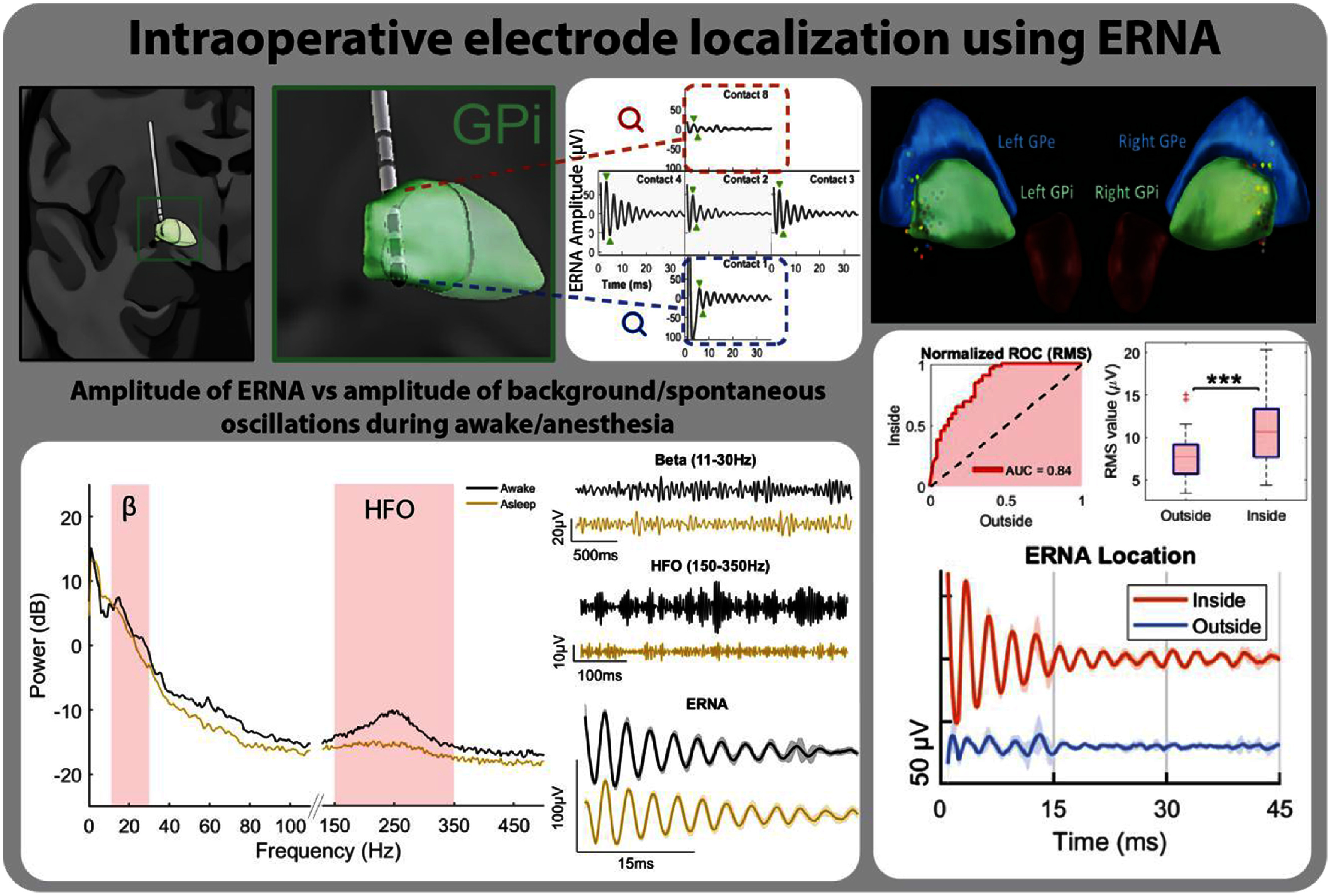
Intraoperative electrode localization using evoked resonant neural activity (ERNA). ERNA is a promising and target-specific biomarker for intraoperative electrode localization during DBS. **Top pannels** Anatomical visualization of the Globus pallidus internus (GPi) alongside example ERNA signals recorded from various contacts on the DBS lead. **Bottom left** panel: local-field potentials (LFPs) recordings during awake and asleep state show robustness against the effects of anesthesia, enabling the potential for asleep DBS procedures [22, 190, 206, 208]. **Bottom right** panel: Spatial and temporal characteristics of ERNA help verify precise targeting. Reprinted from [209], Copyright (2024), with permission from Elsevier.

To enhance the precision of DBS lead placement, recent research has shifted toward intraoperative physiological markers that offer immediate, actionable feedback (figure 5 Anatomic targeting). A key focus has been on intraoperative DLEPs [190], alternatively referred to as ERNA (figure 6) [206–208]. DLEPs (ERNA) are a promising biomarker for STN/GPi targeting optimization, thanks to their higher amplitude compared to LFPs, defined spatial distribution within the STN and GPi, and robustness against the effects of anesthesia, enabling the potential for asleep DBS procedures [22, 190, 206, 208]. DLEPs (ERNA) are associated with a rise in the high-frequency (200–400 Hz) power, which is frequency-dependent and individual for each patient. These factors enable even further optimization of stimulation parameters and improve DBS lead implantation in both awake and under general anesthesia procedures [209, 210]. However, recovering DLEPs (ERNA) remains technically challenging due to stimulation artifacts. To overcome this, new hardware solutions are being developed to suppress these artifacts and allow DLEPs (ERNA) recording even on the stimulation contacts [190, 211]. Current efforts aim to create paradigms for real-time estimation in surgical and clinical decision-making and to examine the effects of different behavioral states or anesthesia on DLEPs (ERNA) dynamics.

#### 4.2.2. Personalized closed-loop DBS for neuropsychiatric disorders

Neuropsychiatric disorders remain difficult to treat because of their diverse presentation across individuals. While the neurobiological underpinnings of these diseases are still being elucidated, neural activity patterns have been associated with specific symptoms [31]. Importantly, alleviation of symptoms through efficacious treatment has led to a concomitant reduction in these ‘neural biomarkers’. Closed-loop neurostimulation is well well-suited therapeutic strategy, as it delivers targeted stimulation only during periods of elevated symptoms as identified by a neural biomarker. Building on this framework, an NIH-funded clinical trial has been established to investigate the use of closed-loop DBS guided by personalized biomarkers and stimulation targets in individuals with major depressive disorder [32, 212]. The ongoing clinical trial includes three stages (figure 5 Sensing): Stage 1—Temporary implantation of SEEG across brain regions associated with depression with testing over several days to identify therapeutic stimulation and biomarker sites; Stage 2—Patients with an identified therapeutic stimulation site are chronically implanted with the NeuroPace RNS System followed by optimization of stimulation and detection parameters; Stage 3—Blinded randomized crossover with three conditions: (1) biomarker controlled closed-loop stimulation, (2) fixed intermittent open-loop stimulation, and (3) sham stimulation to asses efficacy of stimulation. An important consideration for the development of adaptive neurostimulation therapies for neuropsychiatric disorders is the current lack of FDA-approved implantable DBS device with functionality tailored for this purpose. Researchers are therefore limited to using devices designed for epilepsy or movement disorders, which lack one or more crucial capabilities for easy patient use, including: non-tethered data transfer, the ability to change between preprogrammed settings, easy marking of events, and internal clock updating (to accommodate time-zone/daylight saving changes). The functionality of future devices must also be improved for translation into clinical care, with the ability for remote clinical programming and low barrier-to-entry use of recorded data to inform new parameter selection. Next generation devices should be also able to operate in different stimulation modes: continuous open-loop, open-loop with programmable duty cycle, responsive closed-loop, and adaptive stimulation. Each mode should support programmable daily stimulation start times, stop times, or stimulation-on intervals. Lastly, to enable more sophisticated adaptive functionality, devices must be able to synchronize with and integrate information from non-neural data sources, aggregate data to track and act on biomarkers on the scale of hours to days, and incorporate multiple control policies and multidien rhythms into algorithmic control of stimulation delivery. Clinical implementation of these technologies faces several challenges, particularly in the context of intensive neuropsychiatric trial. These include the ability of patients to complete required study activities, clearly delineating the role of study staff in managing medical needs related to the clinical trial but not general psychiatric or medical care, and unknown interaction between pharmacological treatments and neurostimulation. Long-term considerations also remain critical, including ongoing patient support for device programming, decisions around device replacement or explantation, and associated financial responsibilities.

#### 4.2.3. Probing the brain networks using electrical stimulation

Adaptive neuromodulation devices can benefit from measurements that capture brain network state. Single-pulse electrical stimulation is a key technique for studying human brain networks by delivering brief electrical pulses to one region while recording voltage responses in distal regions [213–216]. When stimulating and measuring from cortex, these evoked potentials are referred to as CCEPs. When research includes stimulation targets in subcortical and white matter areas, a more general term, BSEPs, is necessary to describe these measurements. This method is increasingly used to investigate the organization of human brain networks and assess how electrical stimulation modulates functional circuits [217].

Using evoked potential measurements as a biomarker of network function is done in regular clinical practice [213–215]. For example, brainstem auditory evoked potentials are a well-established tool for evaluating the auditory system due to its well-documented anatomical and functional structure [218]. Combining BSEPs with detailed anatomy to characterize the cognitive systems holds potential for developing novel electrophysiological biomarkers for neurological and neuropsychiatric diseases.

Evoked potential biomarkers should characterize specific aspects of brain networks, such as conduction delays that change with development. To characterize how BSEPs change with human brain development, a large cohort study involving 74 patients with implanted iEEG electrodes measured the latency of the initial response in the evoked potential following single-pulse stimulation [219]. Cross-sectional results across at least 20 patients per tracts reveal decrease in latency between endpoints of the arcuate fasciculus, superior longitudinal fasciculus and the temporal-parietal aslant tract. These decreases were observed up to the age of approx. 40 years old, decreasing from *∼*45 ms to *∼*20 ms from 4–40 years old [219]. This developmental trajectory matches development of white matter pathways previously measured with diffusion MRI. These findings indicate that early responses in BSEPs can be used to measure conduction delays in human brain networks.

BSEPs are not only observed in directly connected areas, but may propagate to indirectly connected structures [220]. Hippocampal stimulation, for example, evoked a prominent signal peaking around 200 ms in the posterior cingulate. This response likely propagates along key white matter pathways of the memory and spatial limbic network, including the fornix, mamillary bodies, ANT and the cingulum bundle. This response was observed on a single trial basis. To understand whether such evoked potentials can characterize changes in network excitability, BSEPs were measured before and after patients implanted with iEEG electrodes received a brief trial of neurostimulation therapy, while controlling for changes in electrode impedance [221]. When patients received over 1.5 h of 145 Hz thalamic stimulation the amplitude of BSEPs after stimulation was reduced compared to their baseline BSEPs recordings.

In summary, BSEPs provide valuable insights into anatomical properties and functional state of human brain networks. They may serve as quantitative electrophysiological biomarkers to help understand short-term changes in excitability following neuromodulation protocols [221].

## 5. Technological and engineering considerations

**Figure 7.**
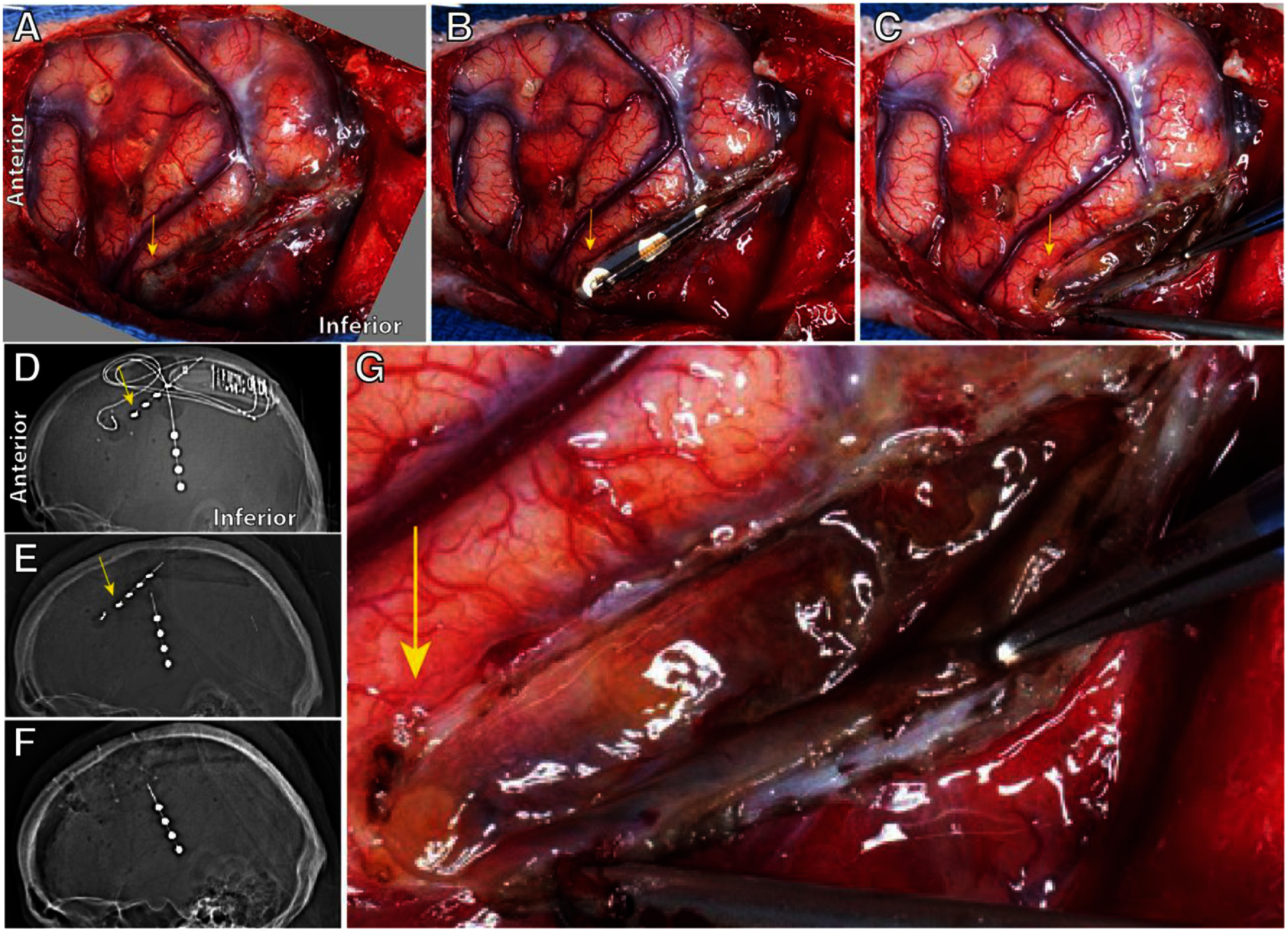
Scarring of tissue after long-term implantation of intracranial devices. Initial implant 4 years prior. (A) Craniotomy before removing the electrode paddle (marked with the yellow arrow). (B) Uncovered paddle electrode. (C) Scarring after the removal of the electrode paddle. (D) Lateral x-ray showing RNS implant at time of presentation. (E) RNS implant was removed in prior craniotomy and replaced with bone putty, but two 4-contact paddles and one depth remained. Strip electrodes could not be slid out from their site of insertion underneath the battery at the time of explant. (F) Lateral x-ray after craniotomy and removal of the paddle electrode. (G) Detailed scarring of the tissue after the electrode removal.

Implantable neurotechnology is increasingly used in clinical practice, entailing products from both established industry leaders and emerging startups (section 2). Yet, the devices most widely utilized are not necessarily the most technically advanced, but those that are easy for clinicians to implement and patients to manage in daily life [10]. For example, aspects such as surgical complications, device replacement and associated tissue encapsulation (figure 7), interoperability between hardware components, or device extrusion impact a surgeon’s decision to employ a certain device. To address this, it is critical to determine the minimum technological requirements needed to deliver meaningful therapy in a simple, safe and cost-effective manner. Translational platforms enable iterative testing and real-time feedback in clinical settings, helping to identify practical system requirements. These findings can inform standard protocols, supporting broader adoption while ensuring patient safety, clinical benefit, and ethical integrity.

### 5.1. Standardization & engineering considerations for neuroadaptive technology

#### 5.1.1. Current standardization efforts

Standardization in the neuromodulation field is highly desirable for lowering the cost of these therapies as well as improving the safety and accessibility for the patients. However, this remains a significant challenge due to the rapidly evolving nature of the technology, which has yet to reach its plateau, and thus there is a lack of consensus on device functionalities and parameters. The need for standardization is driven by three key domains: (1) technology, (2) regulation, and (3) users. While these areas overlap and influence one another, they can generally be distinguished by their primary focus. The *technical* domain primarily addresses functionality, the *regulatory* domain concerns the safety and efficacy of the device, and the *user-oriented* domain focuses on usability and the specific needs of the community using the device.

Current technical standards for neuroadaptive stimulation are governed by the ISO 14708-3s edition *Implants for Surgery—Active IMDs—Part 3: Implantable Neurostimulators* [222, 223]. Regulatory guidance for iBCIs is governed by ”Implanted Brain-Computer Interface Devices for Patients with Paralysis or Amputation—Nonclinical Testing and Clinical Considerations”[224], which primarily serves as a guideline for obtaining IDE. However, there is currently no clear guidance on essential functionalities for which to design or methodologies with which to evaluate iBCIs technologies, especially concerning usability and the fulfillment of user needs [225, 226]. To address this issue, IEEE set up a committee to identify and address gaps in the existing standards. In February 2020, IEEE released a document with a comprehensive overview of the current practices and future requirements for standardization in iBCIs called *Standards Roadmap: Neurotechnologies For Brain-Machine Interfacing* [227]. Several working groups have been established, to emphasize the importance of aligning user needs with human factors engineering, usability engineering, and user-centered design to reduce development costs and enhance device effectiveness, including IEEE P2725.1 [228] (*Standard for Microwave Structural, Vascular or Functional Medical Imaging Device Safety*), IEEE P2794 [229] (*Reporting Standards for In Vivo Neural Interface Research*), and IEEE P2731 [230] (*Standard for Unified Terminology for Brain-Computer Interfaces*). Despite general community support for standardization [231], its implementation remains lagging. Companies with significant market share often resist standardization, fearing that it could enable competitors to gain a technological advantage. The complexity and high entry costs of neuroadaptive technology further limit the emergence of new companies, reinforcing the market position of larger, established companies. Premature over-standardization of the platforms may slow down progress, as the user and technological requirements are still being discovered [232]. However, waiting for technology to reach its limits is not feasible, as the progress usually plateaus when (a) a certain level of therapeutic efficacy has been reached; (b) a technological barrier is hit; (c) a fundamental cost barrier is hit, so that marginal gain in benefit requires a proportional rise in costs. Therefore, it is essential for the neuroadaptive community to anticipate and actively drive standardization efforts. Lastly, legislation needs to be enacted to keep pace with the rapid advancements of neural devices and enforce community-driven standards to prevent misuse and mitigate the risk of market monopolization.

#### 5.1.2. Lessons to be learned from cardiac stimulation field

The first cardiac pacemaker was implanted in Sweden in 1958 [233], commencing clinical cardiac implant procedures that became standard by the mid-1970s [234]. Today, approximately 750 000 pacemakers are implanted annually in the United States alone [233], with devices now offering highly advanced functionalities. The evolution of cardiac pacemakers offers valuable insights into the current state and future potential of neurostimulation technologies. The cardiac field began with fixed rate stimulation for a limited range of indications [234]. Over time, advancements in sensing functionalities have refined stimulation strategies. Nowadays, adaptive stimulation, which aims to preserve physiological activity, is considered gold standard [233]. Similarly, in neurostimulation, fixed stimulation is now an established method, and the field is currently transitioning toward adaptive approaches [16, 17]. Observing the cardiac device development, we can also anticipate further miniaturization of the devices, leading implant procedures to likely become less invasive. Moreover, as the technology matures, we might expect improved computational power, more power-efficient operation and better biomarker design & diagnostic specificity. A trend that is expected to continue is the integration of neural devices into medical applications [84, 155] through wearable WBANs [235]. This integration enables remote monitoring and automated, cloud-based classification of chronic recordings, generating datasets that can be leveraged for training artificial intelligence (AI) classifiers. Unlike cardiac stimulation, neurological pathophysiological mechanisms are much less understood and generally more complex than their cardiac counterparts. This is particularly evident in heterogeneous diseases such as neuropsychiatric disorders, where it remains uncertain whether the exact pathophysiological mechanisms will ever be fully understood. From a regulatory perspective, cardiac implants benefit from robust guidelines covering post-procedural follow-up, optimal device programming, and device selection criteria, as well as extensive studies across large populations and long timeframes [236]. Moreover, since the 1990s, the cardiac field has established unified industry standards, allowing the interchangeability between leads and devices of different manufacturers by implementing IS1/DF1 and later IS4/DF4 standards [237–239].

#### 5.1.3. Practical engineering considerations for device design

When designing implantable devices, engineers primarily focus on optimizing durability and robustness to ensure the device’s longevity. However, devices often require premature removal due to complications, the conclusion of a study, or, as seen in recent years, the issue of abandonment of active implantable neurotechnological devices [240]. Abandoned neurotechnological devices are defined if at least one of the following criteria is met [241]: (1) *Failure to provide information* relevant to plans for medical, technical, and/or financial responsibility during and after the clinical trial. (2) *Failure to fulfill responsibility* for medical, technical, and financial support before the end of the labeled lifetime. (3) *Failure to address any immediate needs* of the patient using the device, which may lead to safety concerns or ineffectiveness of the therapy. (4) *Failure of a clinical research trial* when informed consent failed to address either ongoing access to device management, the possibility of other devices potentially having equal or greater therapeutic effect in the future, or the trial overseer not providing reasonable effort to facilitate continued support for patients who benefit from the device. Regardless of reason, device explantation may pose significant risks, potentially even greater than implantation itself. Cases where craniotomy is needed to fully access and remove components, increases the risk of damaging adjacent nerves, veins, and muscles, heightening the chance of hemorrhage or other brain injury [242]. Moreover, the explant surgery puts a significant financial burden on the patient. The **cost of device explanation** and subsequent **reimplantation** is estimated to be **75505 USD** [243]. Insurance programs generally have no legal obligation to cover device removal unless it is deemed medically necessary for physical reasons (e.g., infection, allergies, device component breakage) [244]. Cosmesis, or the aesthetic appearance of implanted devices, is another critical yet frequently overlooked design consideration. Device visibility directly correlates with the risk of skin erosion, making the cosmetic aspect integral not only to longterm safety but also to patient satisfaction and quality of life [245]. A less visible or well-concealed device may reduce psychological distress and improve daily comfort, ultimately enhancing adherence to therapy. Thus, engineers should prioritize not only the durability but also the explantability and everyday usability of the device. Key areas for improvement include:

• Minimizing surface roughness of components, such as by ensuring paddle electrodes are smooth and rounded, to reduce tissue irritation.
• Enhancing modularity, enabling partial replacements when specific components malfunction, thereby avoiding full explantation.
• Improving cosmetic integration by minimizing extrusion, for example, embedding the device within the skull or relocating it to the chest.

Additionally, advancements in biodegradable materials could offer significant benefits, particularly for short-term therapies or rehabilitation scenarios, such as stroke recovery. Such materials would allow the device to degrade naturally over time, reducing the need to repeat surgeries [246, 247]. Finally, industry and regulatory bodies should establish funding mechanisms to support explantation costs following study completion [248] or in cases of company bankruptcy, thus addressing the issue of orphaned devices [249].

#### 5.1.4. Modularity & Interoperability

One of the primary challenges in neuroadaptive systems is the direct interface between implant components and brain tissue, which becomes encapsulated over time due to gliosis and foreign body response (see figure 7) [18, 250]. Currently, if a system component fails or requires replacement, explantation of the entire device is often necessary, subjecting patients to substantial surgical risks that sometimes exceed those of the initial implantation [243, 245].

Modular implantable systems offer several advantages in this context [231]. By reducing the need for direct re-entry into the brain, they help mitigate risks associated with revision surgeries. Modularity also supports greater personalization by enabling configurations tailored to patient-specific needs, for example, using smaller components for pediatric patients. In the case of localized infections, modular systems may allow for removal of components such as extension cables or leads without necessitating full system explantation. These procedures can often be performed in local hospitals, avoiding referral to specialized surgical centers and facilitating more accessible, targeted treatment [14].

Effective modular design requires alignment with implant location and clinical purpose. In chest-mounted systems, an ideal configuration might include an INS, flexible extensions, and leads consolidated into a single, collapsible cable to reduce mechanical stress points. In skull-mounted systems, modularity may be simplified to a device-and-lead model, allowing the entire system to be embedded within the skull. This can reduce the risk of skin erosion or device extrusion and improve cosmetic outcomes.

One of the most promising areas for standardization in modular systems is connector design [231, 239]. For implantable stimulators with low channel counts (up to 32), low-profile in-line connectors with contact rings have become the design of choice [239]. Major manufacturers such as Medtronic, Abbott, and Boston Scientific use in-line connectors that are interchangeable via extension adapters [46]. However, no formal design standard currently exists, limiting cross-platform compatibility [231, 239]. Companies such as PMT, with its Cortac^®^ grids, and CorTec, with its °AirRay electrodes, are developing cross-platform ECoG solutions that aim to meet both modularity and connector compatibility requirements [251]. These systems are however still undergoing clinical trials and have not yet received regulatory approval for chronic clinical use.

A similar lack of standardization exists in the domain of microelectrode arrays, where no internal connector standard has been established. Standardization in this field is particularly challenging due to high variability in channel counts and array configurations. Although Omnetics connectors are widely used in externalized recording setups and accepted in research environments [252–254], a common standard for implantable high-density systems has not yet emerged.

Nevertheless, certain critical components, such as antennas, chargers, and external controllers, must be subject to strict standardization in the interest of long-term patient safety. These elements are essential for the functionality and durability of the system.

Without regulation, there is a risk that patients may be left with obsolete or unsupported implants when proprietary accessories are discontinued or rendered incompatible.

We argue that the field would benefit significantly from existence of a predefined framework for modularity and interoperability. In a clearly defined and collaboratively agreed-upon framework, innovation could thrive by enabling manufacturers to focus on specific system components. For example, they could develop an INS with embedded biomarker detection and adaptive stimulation without needing to produce leads, batteries, or enclosures, as long as they comply with shared interface standards.

#### 5.1.5. Wireless communication

Another domain for standardization involves wireless communication. The primary methods for wireless communication with IMDs include RF transfer, BT, and NFC [255]. To provide a framework for wireless communication with implantable devices, the MICS frequency band was proposed, along with associated guidelines [256]. However, there is still no standardized media access control address (MAC) protocol designed for MICS networks [256]. Several companies use the MICS band network, with some, like Medtronic Inc. and Biotronik Inc., developing proprietary communication protocols, while others employ the 2.4 GHz ISM (Industrial, Scientific, and Medical) band with established protocols such as Zigbee, BT, or Wi-Fi [256]. Despite existing standards in wireless communication, most companies utilize their own RF receivers to interface with implantable devices with little or no interoperability between the companies. This arises partly from ensuring security and the requirement for receivers to align with the capabilities of the device’s antenna; however, it is more significantly influenced by the fact that companies typically offer complete systems, including proprietary software, controllers, chargers, and other components, rather than just individual modules. The potential for standardization in this domain lies in developing communication modules that are interoperable among different companies. However, this endeavor also brings several challenges, including the necessity for communication modules to accommodate the various functionalities, specific to individual devices, receivers to comply with various frequency bands utilized by implants and security risks, especially when patients are outside controlled medical environments. Potential threats include breaches of confidentiality (unauthorized access to personal information), integrity (unauthorized modification of data in transit), and hostile interactions that could compromise device function [257]. Standardizing secure, interoperable wireless communication protocols could address many of these concerns and improve the usability and safety of implantable devices across various applications.

#### 5.1.6. Standardization of data acquisition, storing and sharing

In recent years, concerted efforts have been directed toward standardizing data management within neurotechnology research and promoting collaborative exchange of tools and resources [258–260]. These initiatives have resulted in the development of accessible repositories and platforms that facilitate the sharing of data, software, and protocols between institutions, accelerating progress in the field.

Notably, initiatives such as OpenMind Consortium and the BRAIN Initiative Alliance Researcher Tools aim to strengthen the neurotechnology research collaboration. These platforms provide access to firmware for device interfacing, data processing software, and practical guidance on setting up and utilizing tools for advanced neuromodulation research.

The large volume of data generated by iEEG recordings (often hundreds of gigabytes per subject) necessitates robust cloud-based storage and sharing solutions. This need is especially critical given the limited number of patients who undergo such procedures, making each dataset valuable for collaborative and reproducible research. Four prominent, freely available data repositories for intracranial neuroelectrophysiology data are the Data Archive for the BRAIN Initiative (DABI) [259], Distributed Archives for Neurophysiology Data Integration (DANDI), OpenNeuro, and Brain-CODE [260].

To ensure data interoperability and reproducibility, standardized data formats have been developed. NWB [261] and the Brain Imaging Data Structure (BIDS) [262] are two widely adopted frameworks designed to unify data and metadata across modalities into a standardized structure. NWB enforces the use of a single data format for electrophysiology [261], improving standardization but increasing conversion complexity. BIDS, by contrast, supports storage in a range of domain-specific formats, including European Data Format and its extensions (EDF/EDF+/BDF), NWB, EEGLab, and MEF3 [262]. Some repositories also accommodate formats like .mat, .csv, .json, HDF5, or BCI2000 .dat files, reflecting diverse priorities in data storage, such as data compression, continuous data acquisition, and event marking. Data sharing protocols align with HL7 standards [263]. Despite progress, inconsistent adherence to best practices in data acquisition continues to be a limiting factor.

An ongoing concern in neurotechnology is data ownership. For recordings collected in research settings, patients must be clearly informed during the consent process about how their data will be used, and the data must be anonymized prior to sharing. In contrast, commercial devices with sensing capabilities regularly collect and store large volumes of data, often capturing aspects of a patient’s mental and emotional state in real time. While such data are essential for ensuring therapeutic efficacy and advancing algorithm development, their collection raises important ethical obligations for companies, particularly regarding data privacy, transparency, and long-term use. For a deeper insight into these issues, readers are referred to the recent work by Soldado-Magraner *et al* [14].

#### 5.1.7. Standardization of intraoperative protocol

With advancements in neuromodulation systems, the need for standardized intraoperative testing protocols has become increasingly critical, particularly in procedures involving awake patients, where behaviorally modulable neural signals must be reliably captured, or in sleep surgeries that rely on disease-specific electrophysiological biomarkers. In these contexts, implementing structured protocols ensures that the devices operate correctly and that stimulation or recording targets are validated in real time. Standardization enhances device effectiveness, improves patient safety, and optimizes surgical workflows by minimizing errors in electrode placement, signal calibration, and stimulation adjustments. It also ensures consistency and reliability across procedures, reducing variability in device performance and enabling meaningful comparisons across clinical studies and trials. These protocols significantly improve device functionality and diagnostic accuracy, though they may slightly extend procedure times initially. Currently, engineers assist during surgeries by performing both passive and active diagnostics of the device and its settings. Passive diagnostics primarily focus on verifying electrode placement, ensuring proper electrode–connector connections, and measuring impedance. In contrast, active diagnostics vary depending on the type of surgery. In awake procedures, they may involve mapping behavioral states, observing disease-specific symptoms, or assessing a patient’s subjective response to stimulation. In sleep surgeries, active diagnostics might include monitoring electrophysiological biomarkers, delivering test stimulations, and evaluating neural responses. By optimizing intra-operative procedures, standardization improves overall accessibility to neuromodulation therapies by ultimately reducing surgical duration and easing demands on medical staff. It also provides a structured framework for training surgical teams, ensuring competency in device handling and testing. Additionally, standardization facilitates the development of technological advancements such as automated calibration systems and remote-controlled testing capabilities, which could improve accessibility for patients in underserved regions. Future advancements in these protocols may further streamline procedures, enhance device precision, and allow for more widespread adoption of neuromodulation treatments while maintaining high safety and efficacy standards.

## 6. Ethical considerations in neuromodulation research

Since the early 2000s, interest in neuromodulation therapies has grown significantly across research and clinical domains [2, 264]. Advances in iBCIs technology, particularly high-profile projects like BrainGate and Neuralink, have drawn widespread public attention [265, 266]. This surge in interest is also accompanied by growing public scrutiny and numerous ethical questions concerning the future of neurotechnology [267, 268]. Moreover, with the increasing volume of research, it is becoming essential for investigators to understand the regulatory landscape of the field. To help orient readers within the complexity of these issues, we provide a brief overview of key regulatory, financial (see sections 2.1 and 2.2 in supplement), and ethical considerations that we believe are most relevant to ongoing and future neuromodulation efforts.

### 6.1. Ethical aspects in neuromodulation research

Invasive neuromodulation raises important ethical concerns because it directly affects the human brain and nervous system. This sensitivity has led to increased public attention and demands for careful oversight. It is essential that clinical trials and technology development in this area are conducted responsibly, with strong ethical standards to protect patient safety and well-being [14]. Expert-led symposiums and discussions play a key role in guiding the ethical direction of the field. At the same time, companies developing these technologies must balance their goal of helping patients with the pressure to generate profit, highlighting the need for transparent and accountable practices.

#### 6.1.1. The role of the patients in discovery and development

Prolonged SEEG monitoring is a standard procedure for patients with epilepsy to guide clinical decision making. Given the significant time patients spend undergoing these recordings, a question arises about how to utilize this time and data most effectively. Potential applications include identifying patient-specific biomarkers, optimizing stimulation paradigms, or exploring network dynamics using single-pulse electrical stimulation to deepen neuroscientific understanding, even in areas not directly associated with patient’s primary diagnosis.

While SEEG monitoring for epilepsy is well-established, its use for other conditions such as depression, schizophrenia, or movement disorders is off-label and subject to regulatory approval. These monitoring sessions provide invaluable data for personalized approaches, including mapping patient-specific biomarkers and identifying stimulation targets. The ultimate goal remains to define universal stimulation targets and optimize clinical therapies. This is particularly challenging for heterogeneous diseases like depression or epilepsy, where multi-node stimulation strategies may be needed, but only sparse sampling of brain regions is available.

Although the field is advancing toward patient-specific therapies, prolonged inpatient monitoring remains labor-intensive and faces scalability challenges due to logistical (e.g. staffing, room availability) and financial (e.g. insurance coverage, balancing research and clinical resources) constraints. Enhancing the support structure while improving patient outcomes is essential for the broader adoption and scalability of these therapies.

#### 6.1.2. The role of the industry

Medical device sales representatives, officially known in some contexts as field clinical engineers, although more commonly referred to as ‘reps’, have become an ubiquitous part of the ORs [269]. However, their presence also brings up a number of ethical, legal, and financial questions [270]. Reps provide essential guidance, ensure the correct use of devices, and contribute to standardizing procedures. However, their involvement also significantly increases the overall cost of these medical technologies, posing economic challenges, particularly for startup companies entering the market, limiting the scalability and broader adoption of innovative medical devices. Technological advances, such as cloud-based systems with AI and remote support, could offer alternatives to in-person reps while optimizing costs. Presence of sales reps in the ORs is prominent mainly in the US healthcare system, due to the economic and historical reasons [234, 271]. In other regions, where healthcare practices differ, hospitals train their staff to manage these devices independently. Although this approach comes with several benefits (reduced costs, enhanced institutional knowledge, reducing dependency on external support), it also puts additional burden on already overloaded staff and without a guarantee of the same level of expertise. While economic constraints and the need for standardization may drive a shift away from sales reps in the ORs, careful planning and cultural adaptation are essential for a sustainable transition. Balancing immediate cost reductions with long-term benefits, such as improved procedural outcomes and increased adoption rates, is crucial for the future of medical device deployment in surgical settings.

## Data Availability

The data that support the findings of this study are available upon reasonable request from the authors.

## Appendix A List of speakers

**Table A1.** Speakers.

Speaker	Title	Topic
Christian Bentler	Cutting-edge Neuroadaptive Implantable technologies—CorTecBrain Interchange platform	Implantable neural devices
Jessica Bowersock (in supplement)	Insights from the STN	DBS
Peter Brunner	Data Integration in Neural Research (Introduction toBCI2000)	Ecosystem for neural research
Timothy Denison	Reflection on Translation Platforms and Ecosystem	Translation of research
Rosana Esteller	Dose Adjustment in Neuromodulation	SCS optimization
Dora Hermes	Electrophysiological biomarkers of direct and indirect connections	Neural connectivity
Jeffrey A. Herron (in supplement)	The Journey of FDA investigational device exemption process for a first-in-human trial with a CorTec Brain Interchange	Regulatory approval
Nuri F Ince	Early Feasibility of Recording iEEG Using BIC System from Human Subjects	Implantable neural devices, DBS
Graham Johnson	Brain State Modeling for Adaptive Closed-loop Neuromodulation	Seizure prediction
Daryl Kipke (in supplement)	High Fidelity Neural Interface Systems	Hardware—electrodes
Christopher K. Kovach (in supplement)	Bringing Experimental aDBS device home	DBS
Vaclav Kremen	Brain Restoration—Intelligent-Stimulation-Ecosystem in Neurology	Ecosystem for Epilepsy patients
Kai J Miller	Protocols for Adaptive Neuromodulation Research in Humans	Ecosystem for neural research
Theoden I. Netoff	Optimizing Neuromodulation therapies: Closing the loop	Stimulation optimization
Enrico Opri	Neuromodulation Therapies in Movement Disorders	DBS
Alexander Rockhill (in supplement)	Identyfing Numerical Cognition Using Stereotactic Electroen-cephalography	Functional mapping
Kristin K. Sellers	Personalized Closed-Loop DBS for neuropsychiatric disorders	DBS
Nathan P. Staff	BCI for amyotrophic lateral sclerosis	BCI Therapy
Ashwin Viswanathan	Neurobiomarkers for DBS in Parkinson’s Disease	DBS
Guangying Wu	NIH Funding and Resources to Support Neuromodulation	Funding

## Appendix B List of attendees

**Table B1.** List of attendees.

Name	Institution	Role
Enes Arslan	Mayo Clinic	Ph.D. candidate
Amir H Ayyoubi	Mayo Clinic	Ph.D. candidate
Matthew R Baker	Mayo Clinic	Postdoc
Patrik Began	Mayo Clinic	Engineer
Christian Bentler	CorTec	Engineer
Behrang F Besheli	Mayo Clinic	Postdoc
Jordan A Bilderbeek	Mayo Clinic	Postdoc
Jessica Bowersock	UofL	Scientist
Luciano R F Branco	Mayo Clinic	Ph.D. candidate
Peter Brunner	Mayo Clinic	Scientist
Timothy Denison	Oxford	Scientist
Derek J Doss	Vanderbilt	Md. Ph.D. candidate
Will Engelhardt	WashU	Engineer
Rosanna Esteller	SSG	Scientist
Matthew Hall	Mayo Clinic	Ph.D. candidate
Dora Hermes	Mayo Clinic	Scientist
Jeffrey A Herron	UW	Scientist
Hossein Heydari	Mayo Clinic	Ph.D. candidate
Harvey Huang	Mayo Clinic	MD. Ph.D. candidate
Michael A Jensen	Mayo Clinic	MD. Ph.D. candidate
Graham W Johnson	Mayo Clinic	Scientist, Surgeon
Panos Kerezoudis	Mayo Clinic	Surgeon, Ph.D. candidate
Daryl Kipke	NNX	Scientist
Bryan Klassen	Mayo Clinic	Physician, Scientist
Christopher K Kovach	UNMC	Scientist
Veronika Krakorova	Mayo Clinic	MD. student
Vaclav Kremen	Mayo Clinic	Scientist
Frederik Lampert	Mayo Clinic	Engineer
Nicholas Luczak	WashU	Engineer
Ghassan S P Makhoul	Vanderbilt	MD. Ph.D. candidate
Kai J Miller	Mayo Clinic	Scientist, Surgeon
Matti Moeller	CorTec	Engineer
Morgan N Montoya	Mayo Clinic	Engineer
Joseph S. Neimat	UofL	Scientist, Surgeon
Theoden I. Netoff	UMN	Scientist
Evan N Nicolai	Mayo Clinic	Postdoc
Gabriela G Ojeda Valencia	Mayo Clinic	Ph.D. candidate
Jhan L Okkabaz	Mayo Clinic	Ph.D. candidate
Enrico Opri	U-M	Scientist
G. Pellizzer	UMN	Scientist
Fiona Permezel	Mayo Clinic	Physician, Scientist
Zeeshan Qadir	Mayo Clinic	Ph.D. candidate
Alexander Rockhill	OHSU	Scientist
Kristin K Sellers	UCSF	Scientist
Nathan P Staff	Mayo Clinic	Physician, Scientist
Chandra Parkash Swamy	Mayo Clinic	Engineer
James Swift	WashU	Engineer
Jamie Van Gompel	Mayo Clinic	Scientist, Surgeon
Ashwin Viswanathan	BCM	Scientist, Surgeon
Gregory A Worrell	WashU	Physician, Scientist
Guangying Wu	NIH	NIH official
Lupita Yanez-Ramos	Mayo Clinic	Postdoc
